# Genetically Directed Production of Recombinant, Isosteric and Nonhydrolysable Ubiquitin Conjugates

**DOI:** 10.1002/cbic.201600138

**Published:** 2016-06-27

**Authors:** Mathew Stanley, Satpal Virdee

**Affiliations:** ^1^ MRC Protein Phosphorylation and Ubiquitylation UnitCollege of Life SciencesUniversity of DundeeDundeeDD1 5EHScotlandUK

**Keywords:** genetic code expansion, isopeptide, oxime, polymerization, synthetic methods, ubiquitylation

## Abstract

We describe the genetically directed incorporation of aminooxy functionality into recombinant proteins by using a mutant *Methanosarcina barkeri* pyrrolysyl‐tRNA synthetase/tRNA_CUA_ pair. This allows the general production of nonhydrolysable ubiquitin conjugates of recombinant origin by bioorthogonal oxime ligation. This was exemplified by the preparation of nonhydrolysable versions of diubiquitin, polymeric ubiquitin chains and ubiquitylated SUMO. The conjugates exhibited unrivalled isostery with the native isopeptide bond, as inferred from structural and biophysical characterisation. Furthermore, the conjugates functioned as nanomolar inhibitors of deubiquitylating enzymes and were recognised by linkage‐specific antibodies. This technology should provide a versatile platform for the development of powerful tools for studying deubiquitylating enzymes and for elucidating the cellular roles of diverse polyubiquitin linkages.

## Introduction

Post‐translational modification of proteins with ubiquitin (Ub) regulates various cellular processes, and defects within this pathway result in numerous pathologies.^1^ Ubiquitylation is orchestrated by a series of enzymes (E1s, E2s and E3s) whose action culminates in the covalent attachment of the Ub carboxy terminus to *N*ϵ‐amino groups of lysine residues by an isopeptide bond.^2^ Ub itself has seven lysines (Lys6, Lys11, Lys27, Lys29, Lys33, Lys48 and Lys63) that can accept another Ub molecule. This results in the formation of seven different isopeptide‐linked polyUb chains, which have been proposed to serve as a cellular code.^3^ Despite the presence of all linkage types in cells, the cellular roles of the various linkages are poorly defined. Furthermore, Ub conjugation is reversible, as the isopeptide bond can be hydrolysed by deubiquitylating enzymes (DUBs), around 100 of which are encoded in the human genome.^4^

Studying the cellular roles of distinct Ub linkages poses a number of challenges because of complications in the preparation of homogenously modified protein and an inability to genetically disrupt distinct linkage types in cells. Nonhydrolysable analogues of polyUb chains of defined linkage type could serve as powerful probes by inhibiting linkage‐specific processes. Assessment of their effects on biological function could help establish their roles.^5^ DUBs are also key cellular regulators and are attractive therapeutic targets. However, the overwhelming number of proteins that associate with DUBs raises the question: which ones are bona fide substrates?^6^ Nonhydrolysable analogues of ubiquitylated substrates could be used as affinity probes to address this question, as DUBs would be predicted to confer specificity for the modified form of the substrate, and the nonhydrolysable nature would circumvent complications associated with DUB‐mediated cleavage of the native counterpart. Nonhydrolysable Ub conjugates of recombinant protein would also facilitate structure determination of substrate–DUB complexes, the raising substrate‐specific antibodies (where native conjugates would be hydrolysed in vivo) and target validation of DUB inhibitors.

Current methods for the nonhydrolysable conjugation of Ub to recombinant protein use a linkage that typically has compromised isostery with the native isopeptide bond (Figure S1 in the Supporting Information).^5^, ^7^ This is often exacerbated by the steric and electrostatic properties of the amino acid scaffolds bearing the requisite reactive handles. Despite a recent refinement^8^ of the cysteine chemistry first reported by Wilkinson and co‐workers,7a this approach still precludes the use of recombinant substrates containing more than one cysteine. Solid‐phase peptide synthesis (SPSS) approaches for preparing nonhydrolysable Ub conjugates have also been described, but these require specialist expertise and are not generally applicable.^9^ Although conjugates formed with triazole‐based linkages have been employed for biological studies,^5^, 7d, 10 the behaviour towards receptors or DUBs that recognise the linkage itself^11^ or that specifically “sense” the inter‐ubiquitin distance in a particular linkage^12^ is unknown. Furthermore, there is no experimental structure of a nonhydrolysable conjugate, and a comparison of the affinity towards DUBs and Ub receptors relative to their native counterparts has not been investigated.

**Scheme 1:**
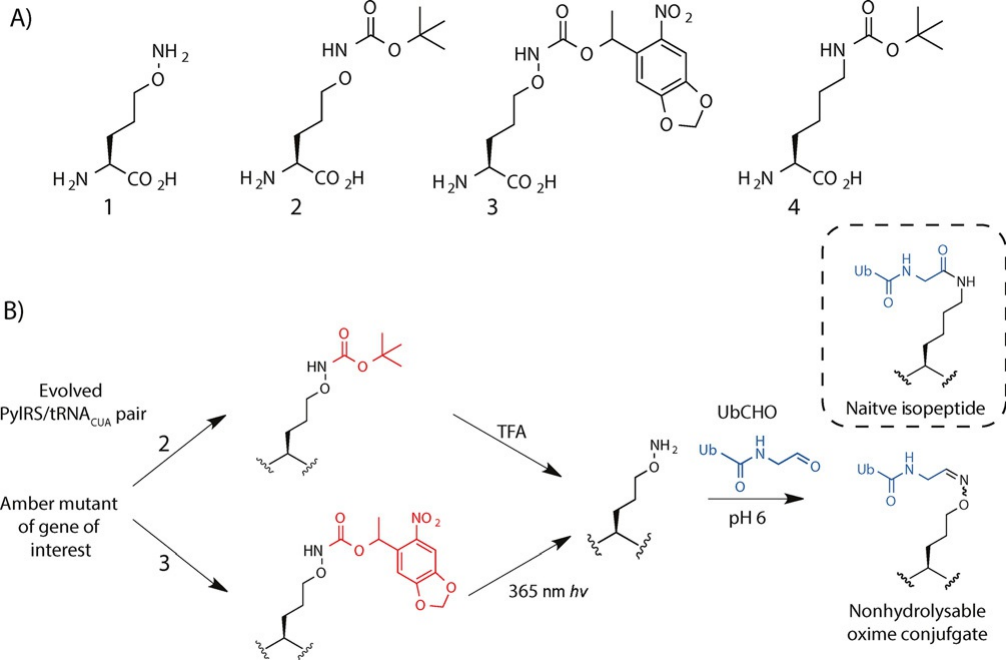
Genetic incorporation of ϵ‐aminooxy‐l‐lysine via acid‐ and photo‐labile precursors. A) **1**, aminooxy‐l‐lysine (aminooxylysine); **2**, *Nϵ*‐(*tert*‐butyloxycarbonyl)aminooxylysine; **3**, *Nϵ*‐photocaged aminooxylysine; **4**, *Nϵ*‐(*tert*‐butyloxycarbonyl)‐ l‐lysine. B) Genetically directing isosteric and nonhydrolysable ubiquitin conjugation. Incorporation of **2** or **3** by an evolved *Mb*PylS/ tRNA_CUA_ pair enables the incorporation of the latent aminooxy functionality. Acidolysis or photolysis provides a facile route to the site‐specific incorporation of **1**. Facile oxime ligation with ubiquitin aldehyde (Ub‐CHO) furnishes a nonhydrolysable isosteric mimetic of the isopeptide bond. The geometry of the oxime conjugate largely reflects that of the native isopeptide counterpart.

Here we report a method for genetically incorporating an aminooxy functionality into recombinant proteins by the incorporation of the unnatural amino acid aminooxy‐l‐lysine (**1**, Scheme 1 A) by using an evolved *Methanosarcina barkeri* (*Mb*) pyrrolysyl‐tRNA synthetase (PylS)/tRNA_CUA_ pair. This enables the site‐specific, nonhydrolysable ubiquitylation of proteins by bioorthogonal oxime ligation.^13^ We demonstrate the generality of this approach by preparing diubiquitin (diUb) of distinct linkage types and SUMO2 (small ubiquitin‐like modifier 2) modified with Ub at a physiologically relevant site. Structural, biochemical and biophysical characterisation of these conjugates revealed that they accurately reflect the topology of their native counterparts. They also serve as potent (nanomolar) DUB inhibitors and provide insight into how the substrate specificity of a ubiquitin carboxy terminal hydrolase (UCH) family DUB towards its substrates is achieved. We also describe a hybrid strategy that involves genetic code expansion and intein chemistry to produce extended nonhydrolysable Ub polymers. This technology should be valuable for the identification of proteins that confer specificity for topologically distinct Ub polymers and ubiquitylated substrates, and also for probing the cellular roles of Ub linkages.

## Results and Discussion

### Genetically encoded ϵ*‐*aminooxy‐l‐lysine for the production of nonhydrolysable ubiquitin conjugates

Simple synthetic peptides containing an aminooxy functionality in place of the ϵ‐amino functionality of a lysine residue can undergo bioorthogonal oxime ligation with Ub carrying a C‐terminal aldehyde group.^14^ This furnishes a stable nonhydrolysable oxime‐linked mimic that has high isostery with the isopeptide bond. However, not only is this approach restricted to synthetic peptides, it also introduces a potentially perturbing unnatural amide linkage within the lysine side chain and disrupts the electronic properties of the Ub C terminus (Figure S1).^14^ Incorporating **1** (Scheme 1 A) by genetic code expansion based on the *Mb* PylS/tRNA_CUA_ pair^15^ would extend this technology to recombinant protein substrates, thereby enabling the production of nonhydrolysable conjugates that have unprecedented isostery with the isopeptide bond (Scheme 1 B, Figure S1). However, we anticipated that it would be challenging to evolve a mutant *Mb* PylS/tRNA_CUA_ pair that could selectively recognise **1** (that differs from native lysine by conservative replacement of the ϵ‐methylene group with an ϵ‐oxygen atom) yet exclude structurally similar and cellularly abundant lysine. Furthermore, a free aminooxy group in the cell could potentially undergo oxime formation with cellular keto compounds such as pyruvate.

**Figure 1:**
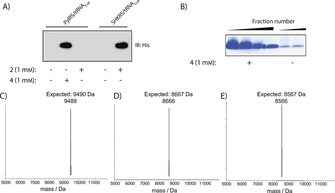
Characterisation of the genetically directed incorporation of **2** at position 6 of ubiquitin. A) *E. coli* cells contained a C‐terminally His‐tagged Ub gene with a TAG codon at position 6, with either the wild‐type *Mb*PylS/tRNA_CUA_ pair or the SHKRS/tRNA_CUA_ pair that contains a Y349W mutation. Cells containing wild‐type *Mb*PylS/tRNA_CUA_ pair produce full‐length protein in the presence of **4**, but failed to incorporate the *N*ϵ*‐*aminooxy analogue **2**. The SHKRS/tRNA_CUA_ pair, which has been shown to incorporate lysine derivatives containing modifications at the δ*‐*position, enabled highly efficient incorporation of **2**, thereby producing full‐length UbBocK6ONH_2_‐His. B) SDS‐PAGE analysis of fractions obtained by Ni‐NTA purification of UbBocK6ONH_2_‐His expressed in the absence or presence of 1 mm **2**. C) Deconvoluted ESI‐MS spectrum obtained from LC‐MS analysis of purified UbBocK6ONH_2_‐His. Observed mass: 9488 Da; expected mass: 9490 Da. D)  His‐tag on UbBocK6ONH_2_‐His was removed by treatment with UCH‐L3. Observed mass: 8666 Da; expected mass: 8667 Da. E) The *N*ϵ*‐*Boc group on **2** was removed by treatment with 60 % aqueous TFA to produce UbK6ONH_2_. Observed mass: 8566 Da; expected mass: 8567 Da.

We considered a latent *N*ϵ‐protected form of **1** that has previously been employed for structurally similar lysine analogues.^16^ The *N*ϵ‐protecting group would also serve as a recognition element for an *Mb* PylS/tRNA_CUA_ pair. The protecting group could then be removed post‐translationally by chemical methods.^17^ Thus we synthesised *N*ϵ‐(*tert*‐butyloxycarbonyl)‐protected aminooxy‐l‐lysine (**2**; Scheme 1 A). Deprotection of the Boc group by acid treatment would furnish **1**. The Boc‐protected derivative was initially chosen, as *N*ϵ‐(*tert*‐butyloxycarbonyl)‐l‐lysine (**4**; Scheme 1 A) is a highly efficient substrate of the wild‐type PylS/tRNA_CUA_ pair.^18^ Derivatives of **4** modified at the neighbouring δ‐position can be incorporated with an evolved SHKRS/tRNA_CUA_ pair that contains a Y349W mutation in the PylS gene.^19^ We therefore tested the abilities of both systems to direct the incorporation of 1 mm **2** into C‐terminally His‐tagged Ub with a TAG codon at position 6.^17^ We found that **2** was not incorporated by the wild‐type PylS/tRNA_CUA_ pair despite, as expected, efficient incorporation of **4** (Figure 1 A). However, the SHKRS/tRNA_CUA_ pair incorporated **2** with efficiency comparable to that of the PylS/tRNA_CUA_ pair with **4** (Figure 1 A). These results verify the first route to genetically direct the incorporation of aminooxy functionality into recombinant proteins, and demonstrate that in the context of the wild‐type PylS/tRNA_CUA_ pair, the Y349W mutation permits incorporation of lysine derivatives augmented at the ϵ‐position as well as the δ‐position.^19^

We next designed and synthesised a photocaged variant of aminooxy‐l‐lysine, **3** (Scheme 1 A). This would allow mild photo‐deprotection of the aminooxy group (Scheme 1 B),^20^ as has been demonstrated in live cells with the analogous lysine derivative.^21^ Not only would this broaden the scope for forming nonhydrolysable Ub conjugates of recombinant proteins in vitro, but it also paves the way for photoactivated bioorthogonal labelling of proteins in live cells. As an evolved PylS/tRNA_CUA_ pair (PCKRS/tRNA_CUA_) has been shown to direct the incorporation of photocaged lysine,^21^ we tested the incorporation of **3** into superfolder green fluorescent protein (sfGFP) with a TAG stop codon at position 150 and a C‐terminal hexahistidine tag,^22^ by using the PCKRS/tRNA_CUA_ pair and a PCKRS/tRNA_CUA_ pair combined with the Y349W mutation (PCKRS*/tRNA_CUA_). Immunoblotting against the His_6_ tag revealed that incorporation of **3** was possible albeit inefficient, and, surprisingly, efficiency was higher with PCKRS (Figure S2). These results indicate that the Y349W mutant only facilitates incorporation of ϵ‐augmentations of lysine derivatives in certain contexts, and alternative mutations can have similar effect, presumably by allosteric restructuring of the active site.

### Production of nonhydrolysable analogues of diUb

**Figure 2:**
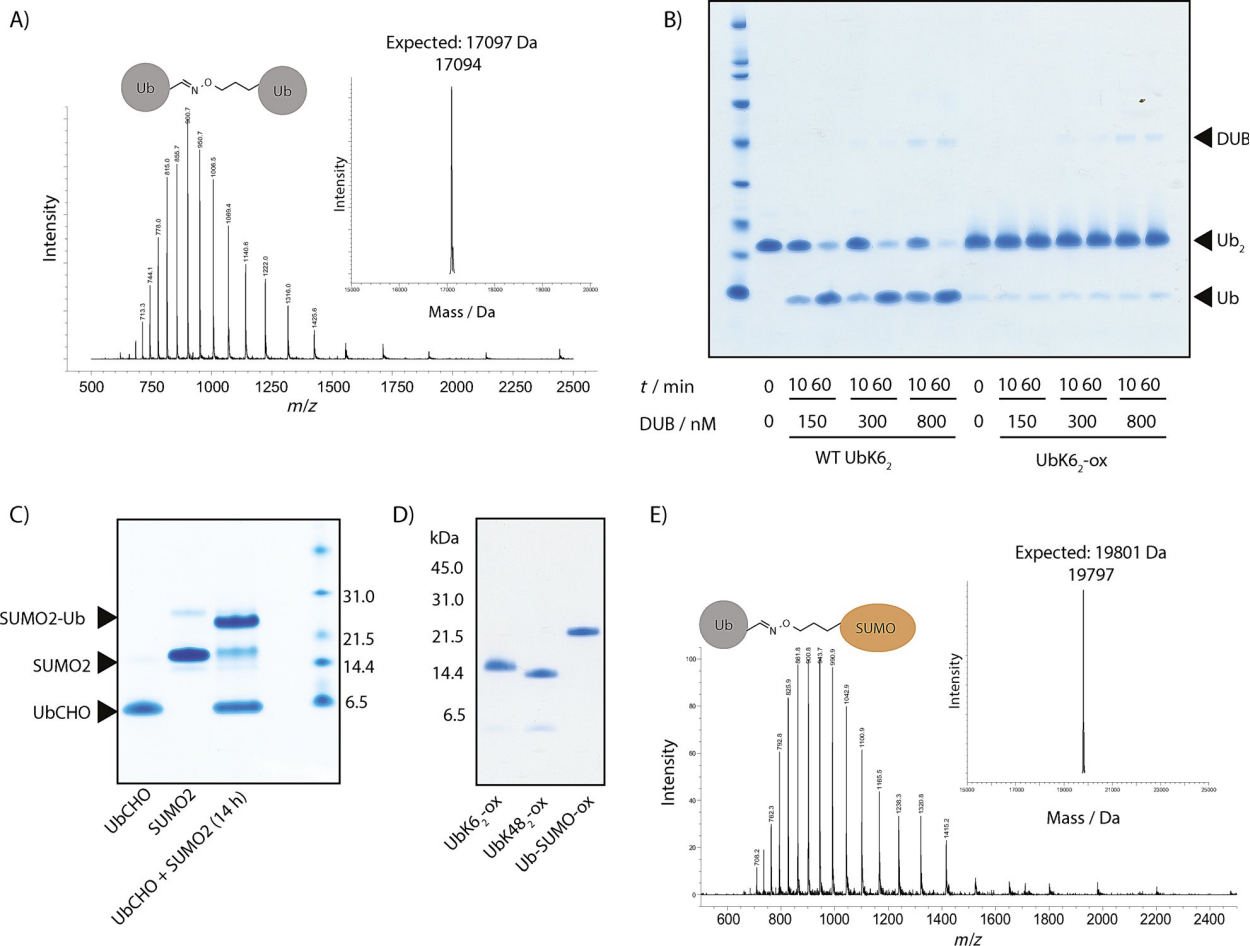
Production of nonhydrolysable oxime conjugates of K6‐linked diubiquitin and ubiquitylated SUMO2. A) ESI‐MS characterisation of the K6‐linked diUb–oxime conjugate (UbK6_2_‐ox). Observed mass: 17 094 Da; expected mass: 17 097 Da. UbK6_2_‐ox was prepared by mixing a twofold excess of Ub‐CHO with UbK6ONH_2_ under denaturing conditions. The product was purified by RP‐HPLC and folded. B) Hydrolytic stability of UbK6_2_‐ox. USP21 is highly active against K6‐polyUb. Native K6‐linked diUb was readily hydrolysed by USP21, whereas UbK6_2_‐ox was completely resistant under the conditions tested. C) SDS‐PAGE analysis of oxime ligation between Ub‐CHO and SUMO2K11ONH_2_. After ligation, the product was purified under native conditions. Reaction catalysed by the addition of 25 mm aniline. D) SDS‐PAGE analysis of the oxime‐linked conjugates: UbK6_2_‐ox, UbK48_2_‐ox and Ub‐SUMO‐ox. E) ESI‐MS characterisation of Ub‐SUMO‐ox. Observed mass: 19 797 Da; expected: 19 801 Da.

We expressed and purified 3.5 mg of His‐tagged Ub containing **2** at position 6 (Ub‐BocONH_2_K6; Figure 1 B), and characterised it by ESI‐MS (Figure 1 C). The C‐terminal His‐tag was removed by treatment with the DUB UCH‐L3,^17^ then Ub‐BocONH_2_K6 was purified by reversed‐phase (RP)‐HPLC (Figure 1 D). The Boc protecting group was subsequently removed by treatment with 60 % TFA to yield Ub‐ONH_2_K6, and the polypeptide was recovered by ether precipitation^17^ (Figure 1 E). In parallel, we prepared Ub aldehyde (Ub‐CHO) as described previously.^23^ Incubation of Ub‐ONH_2_K6 with a twofold excess of Ub‐CHO in denaturing buffer for 1 h at pH 6 resulted in formation of the site‐specifically conjugated diUb product (UbK6_2_‐ox); this was purified by RP‐HPLC, refolded and characterised by ESI‐MS and SDS‐PAGE (Figures 2 A, D and S3). Resistance to DUB hydrolysis was confirmed by treatment with increasing concentrations of the DUB USP21^24^ (Figure 2 B). We observed no hydrolysis of UbK6_2_‐ox at 37 °C for 1 h (even with 800 nm enzyme), whereas native K6‐linked diUb exhibited near complete hydrolysis in the presence of 800 nm USP21 after 1 h (Figure 2 B). Preparation of K48‐linked diUb was also carried out by expression of Ub containing a TAG codon at position 48 (Figures 2 D and S3).

### Production of nonhydrolysable analogues of ubiquitylated SUMO

In order to explore the generality of preparing nonhydrolysable Ub conjugates, we prepared a nonhydrolysable isostere of native SUMO ubiquitylated at Lys11 (Ub‐SUMO2K11). As SUMO2 has a native cysteine, this would test the compatibility of our strategy with cysteine‐containing proteins. Arsenic‐induced formation of Ub‐SUMO2K11 on promyelocytic leukaemia protein (PML) leads to resolution of acute promyelocytic leukaemia (APL).^25^ The physiological DUB that reverses this conjugation is unknown. Intriguingly, DUBs belonging to the Ub C‐terminal hydrolase (UCH) family,^4^ which have historically been considered inactive towards ubiquitylated proteins, have recently been shown to have high activity towards Ub‐SUMO2K11.^26^ As the mechanism of this activity is unknown, an isosteric yet nonhydrolysable analogue of Ub‐SUMO2K11 would be valuable for identifying DUBs that remove Ub from PML conjugates, as well as a structural tool for determining the activity requirements of UCH‐family DUBs. We explored the possibility of carrying out the production of oxime‐linked Ub‐SUMO2K11 (Ub‐SUMO‐ox) on folded protein, without chaotropic salts, by using a one‐pot deprotection–ligation strategy. His‐tagged SUMO2 bearing **2** at position 11 (SUMO‐BocONH_2_K11) was expressed in good yield (3 mg L^−1^ culture medium; Figures S4 and S5). After purification, acid deprotection of the Boc group was carried out on the folded protein.^27^ Quantitative removal of the Boc group was confirmed by LC‐MS (Figure S6). The pH was then raised to pH 7, and a twofold excess of Ub‐CHO was added. Aniline‐catalysed oxime ligation^28^ at 37 °C was then monitored by LC‐MS. The reaction went to near completion after 15 h (Figure 2 C), and the oxime‐linked conjugate (Ub‐SUMO‐ox) was purified under native conditions by sizeexclusion chromatography and characterised by SDS‐PAGE and ESI‐MS (Figures 2 D, E and S3).

### Structural characterisation of oxime‐linked diUb

**Figure 3:**
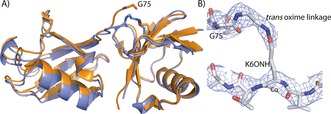
Structural characterisation of ubiquitin K6‐linked oxime conjugate by X‐ray crystallography. A) The 3.5 Å structure of UbK6_2_‐ox (blue) superimposed on the crystal structure of native isopeptide‐linked K6 diUb (orange): backbone RMSD 1.1 Å. B) The aminooxylysine amino acid at position 6 (K6ONH_2_) of the proximal Ub molecule, oxime‐linked to the C terminus of the distal Ub. The mesh corresponds to the 2Fo‐Fc electron density map contoured at 1.0σ. This reveals that the oxime linkage is the *trans* regioisomer.

In order to unequivocally demonstrate that the oxime conjugates accurately mirrored the structure of native isopeptide‐linked conjugates we solved a crystal structure of UbK6_2_‐ox. The crystal structure of native K6‐linked diUb (UbK6_2_) has been determined, and this served as a reference to assess the isostery of the oxime‐linked counterpart.^17^ UbK6_2_‐ox readily formed cubic crystals under identical conditions to those employed for UbK6_2_, thus enabling determination of a 3.5 Å crystal structure (Figure 3 A and Table S1). The topology of UbK6_2_‐ox accurately mirrored that of native UbK6_2_ (backbone RMSD 1.1 Å). An important consideration with our strategy is that the oxime linkage could potentially form a mixture of *cis* and *trans* regioisomers, thereby giving rise to structural heterogeneity.^29^ However, unambiguous electron density for the carboxy terminal residues of the distal Ub molecule and the oxime linkage with incorporated **1** was consistent with the *trans* regioisomer (Figure 3 B). We cannot exclude the possibility that a fraction of the *cis* isomer was present, and that the *trans* species selectively crystallised under the conditions tested. However, we suspect that the steric bulk of the protein reactants ensures that the favoured regioisomer upon oxime ligation is the *trans* species. These findings established that the topology of oxime‐linked conjugates is homogenous and near identical to that of the native counterpart.

### Nonhydrolysable oxime‐linked Ub conjugates are potent DUB inhibitors and bind with affinity comparable to that of native conjugates

**Figure 4:**
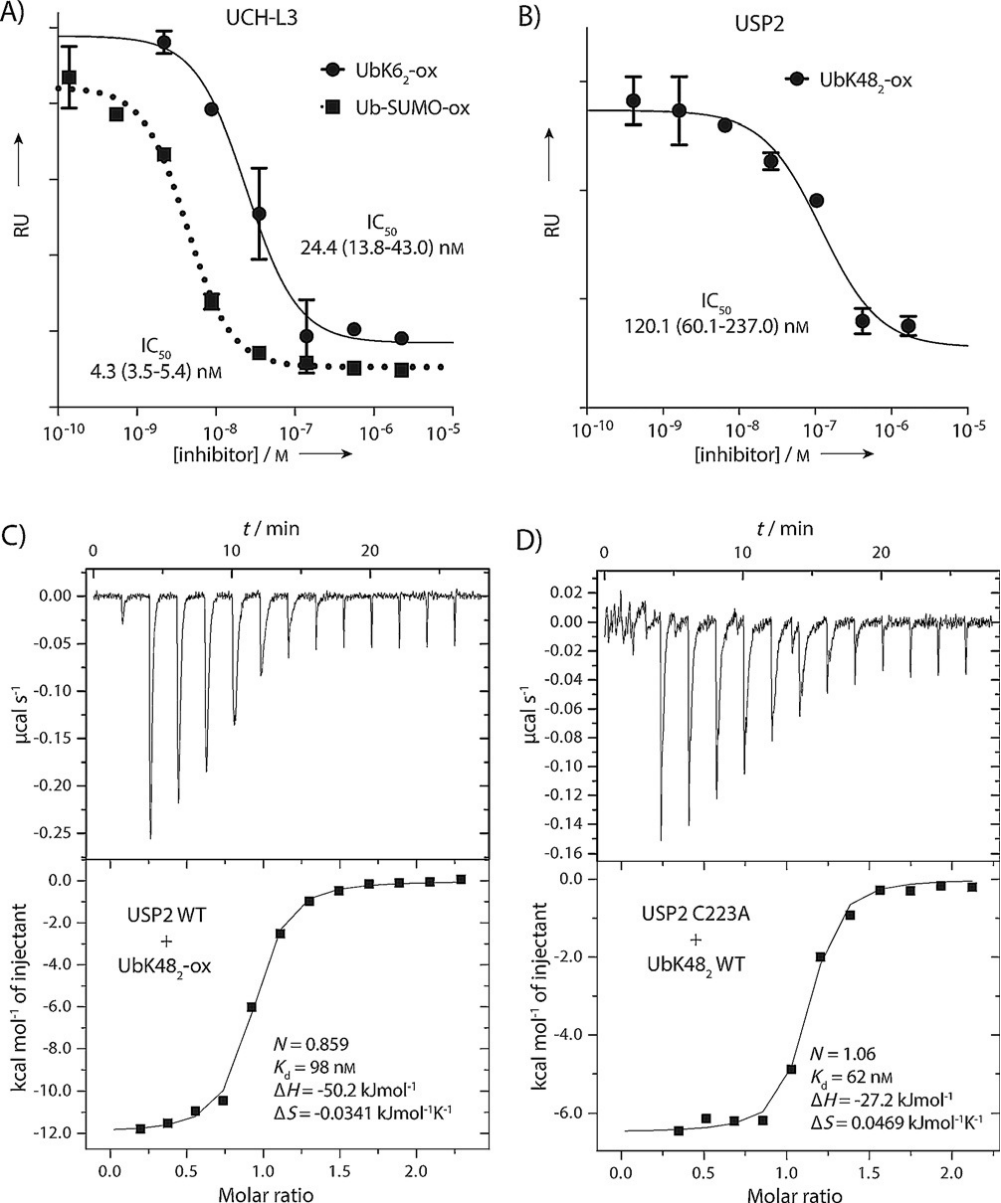
Oxime‐linked ubiquitin conjugates are potent DUB inhibitors and have comparable affinity to native isopeptide‐linked ubiquitin conjugates. A) UbK6_2_‐ox and Ub‐SUMO‐ox potently inhibit the ubiquitin C‐terminal hydrolase (UCH) DUB UCH‐L3 (mean±SD, *n*=2). Native ubiquitylated SUMO is a substrate of UCH‐L3, whereas UbK6_2_ is not. This suggests that discrimination of these substrates by UCH‐L3 is achieved only by differences in *k*_cat_. RF: relative fluorescence units. B) The ubiquitin‐specific protease (USP) DUB USP2 is inhibited by UbK48_2_‐ox. C) ITC demonstrates that *K*_d_ is comparable to IC_50_. Binding stoichiometry is also close to unity, thus indicating that the oxime‐linked conjugate confers inhibition in the IC_50_ assays. D) *K*_d_ of binding between native UbK48_2_ and a catalytically inactive USP2 mutant (USP2 C223A) is comparable to that of UbK48_2_‐ox binding to wild‐type USP2.

We next determined if the oxime‐linked conjugates recapitulated the biochemical properties of the native isopeptide‐linked conjugates, by measuring their capacity to inhibit DUBs. For this we determined IC_50_ values against hydrolysis of the fluorogenic substrate Ub‐Rhodamine.^30^ The conjugates Ub‐ox‐SUMO and UbK6_2_‐ox inhibited hydrolysis of Ub‐Rhodamine by GST‐tagged UCH‐L3 (UCH‐L3; IC_50_: 4.3 (2.5–5.4) and 24.4 (13.8–43.0) nm, respectively; Figure 4 A). As both conjugates were potent inhibitors of UCH‐L3 but only Ub‐SUMO2K11 is a substrate, UCH‐L3 activity is not dictated by *K*_m_, but rather by the significantly enhanced catalytic efficiency (*k*_cat_) towards Ub‐SUMO2K11. We also tested the inhibitory capacity of UbK48_2_‐ox against the USP family DUB, USP2 (Figure 4 B): UbK48_2_‐ox inhibited USP2 with an IC_50_ of 120.1 (60.1–237.0) nm. In the inhibitory assays, the DUB concentrations were extremely low (<2.4 nm); therefore, in order to unequivocally confirm that inhibition was achieved by the oxime‐linked conjugates (rather than trace contamination with Ub‐CHO, a known DUB inhibitor),^31^ we determined the dissociation constant (*K*_d_) between USP2 and UbK48_2_‐ox by isothermal titration calorimetry (ITC; Figure 4 C). *K*_d_ was 98 nm (comparable to IC_50_)_,_ and the binding stoichiometry was close to unity (0.86), thus confirming that UbK48_2_‐ox was indeed the inhibitory species in the IC_50_ assay. In order to demonstrate that the binding affinity of UbK48_2_‐ox recapitulated that of the native conjugate, we characterised the binding between UbK48_2_ and the catalytically inactive USP2 mutant, C223A (Figure 4 D). The dissociation constants were comparable (62 and 98 nm). Although the thermodynamic signature was distinct, it is common for minor structural perturbations to give rise to significant enthalpy–entropy compensation effects without any gross change in binding mode.^32^

### Immunoblotting of wild‐type ubiquitin dimers and oxime conjugates

**Figure 5:**
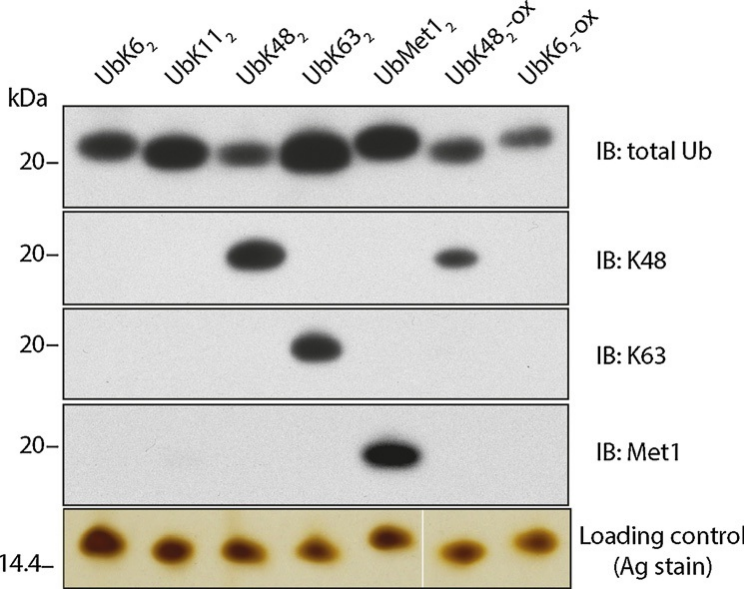
Immunoblotting analysis of native isopeptide linked ubiquitin dimers compared to oxime‐linked ubiquitin conjugates. A comparison of oxime‐conjugated ubiquitin dimers (UbK48_2_‐ox and UbK6_2_‐ox) to wild‐type ubiquitin dimers (UbK6_2_, UbK11_2_, UbK63_2_, UbK48_2_) indicates that oxime‐conjugated ubiquitin dimers are recognised similarly by an α‐Ub (total Ub) antibody. Immunoblotting with linkage‐selective α‐Ub antibodies (α‐K48, α‐K63 and α‐Met1) indicates that UbK48_2_‐ox was successfully recognised by the linkage specific α‐K48 antibody. No cross‐reactivity with α‐K63 or α‐Met1 was observed for UbK6_2_‐ox or UbK48_2_‐ox; only the relevant wild‐type Ub conjugates were recognised. Silver staining was used as a loading control because of inconsistent immunoreactivity of total Ub antibodies across different linkages types.

Recognition of the oxime conjugates by Ub linkage‐specific antibodies against the native counterparts would provide further validation of the physiological integrity of our conjugates. Furthermore, this would validate using the nonhydrolysable analogues as antigens (thereby capitalising on their enhanced in vivo half‐life) for raising Ub linkage‐specific antibodies that could be used in a reciprocal manner to specifically recognise the native isopeptide‐linked conjugate. Linkage‐specific antibodies for K6, K27, K29 and K33 linkages are currently unavailable but would be powerful tools for elucidating the cellular roles of these linkage types. The diUb oxime conjugates (UbK48_2_‐ox and UbK6_2_‐ox) were analysed by immunoblotting with linkage‐specific antibodies along with Ub dimers with native isopeptide linkages (UbK6_2_, UbK11_2_, UbK63_2_ and UbK48_2_; Figure 5). The K48 linkage‐specific antibody^33^ recognised UbK48_2_‐ox, albeit with a slightly weaker signal than for the native counterpart (Figure 5). Furthermore, as expected, anti‐K63^33^ and anti‐Met1^34^ linkage‐specific immunoblotting did not cross‐react with either of the oxime‐linked Ub conjugates (Figure 5). Additionally, total Ub immunoblotting indicated that UbK6_2_‐ox and UbK48_2_‐ox were recognised to the same degree as their respective native isopeptide conjugates, thus indicating that the oxime linkage did not give rise to aberrant masking of functional epitopes on the Ub surfaces or to alteration in the topology of the Ub conjugate (Figure 5).

### Nonhydrolysable polyUb conjugates by oxime polymerisation

Ub chains often elicit a biological response only when present as extended polymers.^35^ We hence established a strategy for the facile preparation of oxime‐linked polymeric Ub. We envisioned that the incorporation of **1** site‐specifically into Ub bearing a C‐terminal aldehyde moiety would produce a molecule that could undergo self‐polymerisation and provide a convenient route to extended oxime‐linked Ub polymers.^5^, ^36^

**Figure 6:**
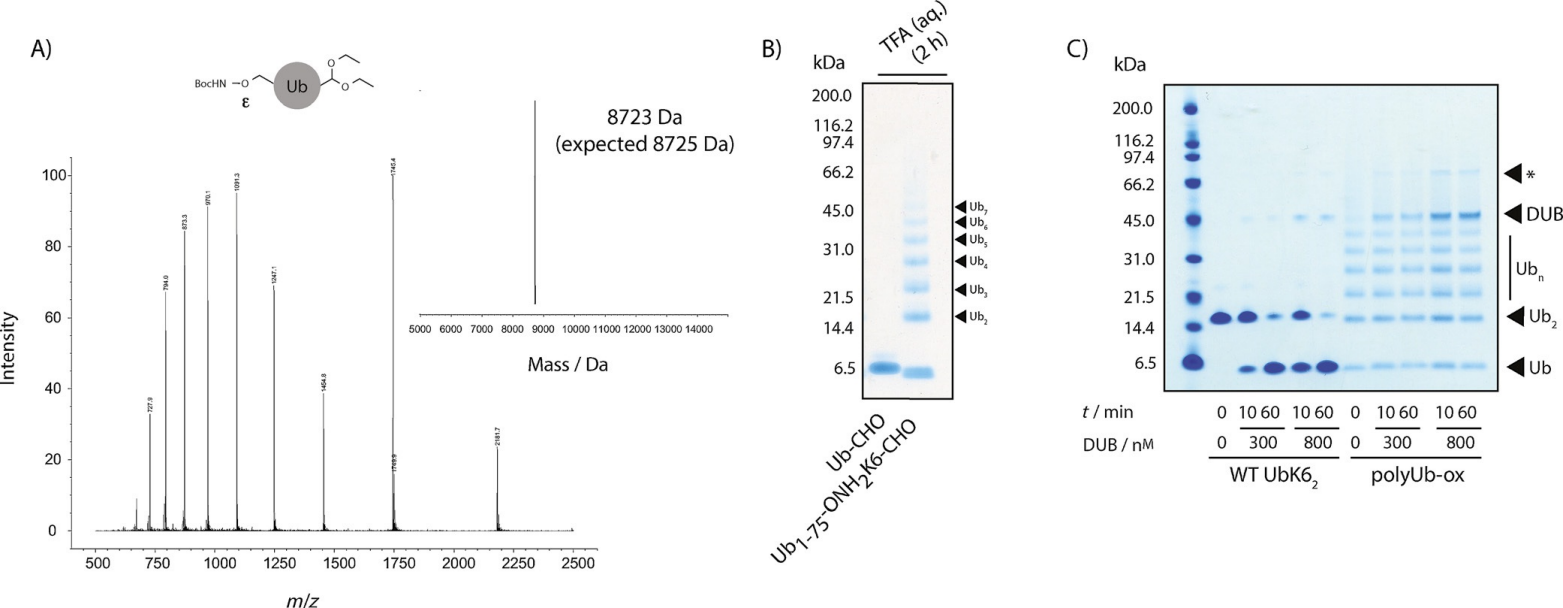
Upon deprotection, the produced bifunctional Ub molecule, Ub_1–75_‐BocONH_2_K6‐acetal can undergo oxime polymerisation to generate nonhydrolysable polyubiquitin conjugates. A) LCMS analysis showing successful synthesis of the protected bifunctional Ub monomer, Ub_1–75_‐BocONH_2_K6‐acetal, for generating oxime‐linked polyubiquitin conjugates (observed mass: 8723 Da; expected: 8725 Da). B) SDS‐PAGE analysis of the TFA‐mediated oxime polymerisation reaction of Ub‐CHO (control) and Ub_1–75_‐ONH_2_K6‐CHO. C) Hydrolytic stability of polyUb‐ox against USP21. Native K6‐linked diUb was readily hydrolysed by USP21 whereas polyUb‐ox was completely resistant under the conditions tested. *: contaminating species in DUB preparation.

In order to prepare the requisite bifunctional Ub monomer, we genetically encoded precursor **2** into Ub resides 1–75 expressed as an intein fusion protein. Thiolysis of the fusion with sodium mercaptoethanesulfonate (MESNa) generated a truncated Ub thioester bearing **2** at position 6 (Ub_1–75_‐BocONH_2_K6‐SR), which was obtained in excellent yield (5 mg L^−1^) and purified by RP‐HPLC (Figure S7). Direct aminolysis of Ub_1–75_‐BocONH_2_K6‐SR with aminoacetaldehyde diethylacetal^23^ yielded Ub_1–75_BocONH_2_K6‐acetal with latent bifunctionality (Figure 6 A). As a control, an equivalent species containing **4** was prepared (Ub_1–75_‐BocK6‐acetal; Figure S8).

Deprotection of the C‐terminal diethylacetal and the Boc protecting groups by incubation in 60 % aqueous TFA resulted in the generation of oligomeric oxime‐linked polymers (polyUb‐ox; Figure 6 B). Ether precipitation of the polymerised material was followed by protein folding and its analysis by SDS‐PAGE (Figure 6 B). In a control reaction, Ub_1–75_‐BocK6‐acetal did not generate polyUb species, thus indicating that non‐specific imine formation does not occur under these conditions (Figure 6 B). The polymers were resistant to DUB hydrolysis, as expected (Figure 6 C).

These nonhydrolysable Ub polymers would be valuable tools for affinity purification of linkage‐specific DUBs and ubiquitin‐binding proteins from cell extracts. Thus, we conjugated polyUb‐ox to biotin, thereby enabling immobilisation on streptavidin resin, by site‐specific C‐terminal thiazolidine formation with cysteine‐functionalised biotin (Cys‐biotin; Figure S9).^37^

## Conclusion

We describe a powerful toolkit based on oxime ligation to advance the study of substrate‐specific DUBs and the cellular roles of polyUb linkages, based on the first genetically directed incorporation of aminooxy functionality into recombinant proteins. This was achieved by the incorporation of either acid‐ or photo‐labile protected precursors of aminooxy‐l‐lysine (**1**). Amino acid **1** has extremely high isostery with native lysine as it differs by conservative substitution of a methylene group by an oxygen atom at ϵ‐position. We demonstrated that **1** could be site‐specifically incorporated into both Ub and SUMO2 via a protected precursor. This enabled the chemoselective conjugation of Ub with Ub‐CHO by oxime ligation. The dimeric Ub conjugates were resistant to DUBs that are highly active against the native isopeptide‐linked counterparts. The oxime linkage exhibits unprecedented isostery with the native isopeptide bond, thus making it a preferred strategy for preparing nonhydrolysable versions of Ub conjugates. This was demonstrated by structural characterisation of a K6‐linked Ub dimer by X‐ray crystallography. The structure of the oxime‐linked species accurately mirrored that of the native counterpart. Furthermore, unambiguous electron density confirmed that the *trans* regioisomer was the predominant, if not exclusive, product upon oxime ligation between proteins. The nonhydrolysable oxime‐linked Ub conjugates also proved to be nanomolar DUB inhibitors. This high isostery with native conjugates, combined with hydrolytic stability, should allow ubiquitin conjugates prepared by this approach to be used as inhibitors of linkage specific processes. Such experiments could be conducted with cell extracts or in intact cells by microinjection. As functionalisation of Ub‐like (Ubl) proteins^38^ with an aldehyde group is possible,^23^ it should also be possible to prepare nonhydrolysable variants of Ub‐like conjugates (e.g., NEDD8, ISG15, SUMO).

Furthermore, we describe the use of oxime chemistry in polymerisation reactions with bifunctionalised Ubs, in order to generate polyUb conjugates linked by oxime isopeptide isosteres. The expedient synthesis of such conjugates, in conjunction with their resistance to proteolytic hydrolysis, makes these new conjugates important probes for studying cellular processes that are regulated by polyUb chains.

Finally, we described the incorporation of photocaged aminooxy‐l‐lysine (**3**). This should broaden the utility by enabling conjugation to acid‐sensitive recombinant proteins. Although incorporation efficiency was low, a more efficient PylS/tRNA_CUA_ should be obtainable by directed evolution.^39^ Furthermore, recent reports have demonstrated that the aminooxy group can undergo rapid biocompatible oxime ligation with dialdehyde moieties^40^ and in boronic‐acid‐mediated oxime ligations.^41^ These reactions are ultra‐fast, rivalling state‐of‐the‐art inverse electron‐demand Diels–Alder bioconjugation between tetrazines and strained enes.^42^ This would enable ultra‐fast photoactivated protein labelling, thereby overcoming the diffusion limit associated with constitutively reactive bioorthogonal handles. Incorporation of the photocaged amino acid into proteins in live mammalian cells should be achievable, as this has been shown for structurally similar, photocaged lysine derivatives.^21^, ^43^ Furthermore, aldehyde functionality can be genetically encoded by using orthogonal aldehyde tag technology (compatible with prokaryotic and mammalian hosts).^44^ Excitingly, this could provide a strategy for site‐specific photoactivated covalent protein–protein tethering in live cells.

## Experimental Section

**Production of K6‐linked and K48‐linked diubiquitin by oxime ligation**: Using previously reported plasmids, UbBocONH_2_K6‐His and UbBocONH_2_K48‐His (UbBocONH_2_KX) were expressed in BL21(DE3) cells supplemented with **2**, followed by His‐tag cleavage.^17^ Boc‐protecting groups were removed on RP‐HPLC‐purified material (2 mg, 233 nmol) by using cold 60 % aqueous TFA (400 μL) at 23 °C for 2 h. Deprotected UbBocONH_2_KX (UbONH_2_KX) was then ether precipitated. Lyophilised Ub‐acetal^23^ (4.0 mg, 468 nmol) was dissolved in denaturing buffer (Na_2_HPO_4_ (200 mm, pH 6) with GdnCl (6 m)), then the pH was then adjusted to pH 3 with HCl (1 n). Acetal hydrolysis for 1 h at 37 °C generated Ub‐CHO. The pH was then raised to pH 6 with NaOH (4 n) and the solution was then used to dissolve precipitated Ub‐ONH_2_KX. Oxime ligation (37 °C for 1 h) followed by semi‐preparative HPLC (20–50 % CH_3_CN+0.1 % TFA, over 45 min) afforded UbKX_2_‐ox, which was lyophilised and refolded by dissolution in Na_2_HPO_4_ (200 mm, pH 7.5) with GdnCl (6 m) followed by overnight dialysis against PBS (UbK6_2_‐ox: 2.4 mg, 60 % yield; UbK48_2_‐ox: 3.4 mg, 85 % yield).

**SUMO–Ub ligation**: SUMO‐BocONH_2_K11 was expressed from plasmid pCDF‐PylT‐SUMO2TAG11‐His_6_ 19 and purified by Ni‐NTA chromatography. Fractions containing SUMO‐BocONH_2_K11 were pooled and concentrated to ∼2 mg mL^−1^_._ An aliquot (600 μL, ∼1.2 mg, 106 nmol) was chilled on ice, then TFA (25 μL) was added. The solution was incubated at 37 °C for 4 h, at which time LC‐MS analysis indicated quantitative deprotection. The reaction was neutralised (pH 7) by addition of NaOH (4 n). Ub‐CHO (625 μL, 2.9 mg mL^−1^, 212 nmol) and aniline (25 mm) was then incubated for 10 min at 37 °C. Deprotected SUMO solution was added to the Ub‐CHO/aniline solution (SUMO/Ub‐CHO, 1:2), incubated at 37 °C for 15 h, with the reaction monitored by LC‐MS. The solution was then concentrated to 0.5 mL in 0.5 mL Amicon centrifugal filters (3 KDa MWCO) and was purified by size‐exclusion chromatography with PBS as running buffer and a Superdex column (S75, 16/60; GE Life Sciences). Subsequent concentration of the appropriate fractions in 15 mL Amicon centrifugal filters (3 KDa MWCO) gave Ub‐SUMO‐ox (1 mg mL^−1^, 36 % yield).

**Crystallisation, structure determination and refinement**: UbK6_2_‐ox crystallised with cubic morphology at 0.8 mg mL^−1^, under previously reported conditions.^17^ The best crystal diffracted to 3.5 Å (Beamline I02, Diamond Light Source, Harwell, UK). Initial phases were obtained by molecular replacement by using one ubiquitin moiety from the K6‐diubiquitin structure (PDB ID: 2XK5).^17^ Indexing and integration was carried out with XDS.^45^ Structure refinement was carried out with PHENIX^46^ and model building was carried out within COOT.^47^ Data collection and refinement statistics are in Table S1 (PDB ID: 5KHY).

**IC_50_ determination**: DUBs, prepared as previously reported,^26^ were diluted (GST‐UCH‐L3: 96 pm; USP2: 2.4 nm) in activation buffer (Tris**⋅**HCl (25 mm, pH 7.5), NaCl (150 mm), DTT (10 mm), BSA (0.1 mg mL^−1^)). A 1:4 dilution series of the oxime‐linked conjugates was then prepared in assay buffer (Tris**⋅**HCl (50 mm, pH 7.5), NaCl (50 mm), DTT (5 mm), BSA (0.1 mg mL^−1^)): UbK6_2_‐ox: 4.5 μm–4.4 nm; Ub‐ SUMO‐ox: 4.5 μm–270 pm; UbK48_2_‐ox, 3.3 μm–814 pm. DUBs (2.5 μL) were dispensed into a low‐volume 384‐well plate (Corning #3676) followed by the dilution series of the oxime‐linked conjugate (2.5 μL). A replicate series was also dispensed. The plate was incubated at 23 °C for 15 min, then Ub‐Rho (2.5 μL, 600 nm in assay buffer) was added. After 15 min, end point measurements were taken in a PherastarFS plate reader (BMG Labtech) fitted with a 480/520 nm fluorescence intensity module. Data were fitted to a four‐parameter dose–response curve in Prism 6 (Graphpad Software, La Jolla, CA).

**Isothermal titration calorimetry**: ITC was conducted on an iTC200 Microcalorimeter (GE Healthcare). All proteins were dialysed against argon‐purged ITC buffer (20 mm HEPES, 100 mm NaCl, 0.25 mm TCEP, pH 7.5). The sample cell contained either UbK48_2_ WT (9.2 μm) or UbK48_2_‐ox (8.4 μm); the concentrations were determined by using a standard curve of UbK48_2_ (absorbance at 214 nm). The syringe contained USP2 WT (93 μm) or USP2 C223A (99 μm); the concentrations were determined by absorbance at 280 nm (for USP2; ϵ_280_=41 370 L mol^−1^ cm^−1^). In all, 13 injections (0.4 μL) were delivered (0.8 s addition time, interval 120 s). The stirrer speed was set to 750 rpm, and each binding experiment was carried out at 25 °C. Water was used in the reference cell, and titrations into buffer were carried out to assess the enthalpies of dilution. The data was smoothed with the simple arithmetic function in the Origin 7 data analysis software (v.7.0552; OriginLab, Northampton, MA).

**Generation of Ub_1–75_‐BocONH_2_K6‐acetal**: The previously reported plasmid pTXB1‐Ub1‐76^17^ underwent two rounds of site‐directed mutagenesis to install a TAG stop codon at Lys6 and to delete the codon for Gly76, thereby generating pTXB1‐Ub1‐75TAG6. pTXB1‐Ub1‐75TAG6 was transformed into *Escherichia coli* ER2566 cells (NEB) to express Ub_1–75_‐BocONH_2_K6‐SR, which was isolated by chitin‐resin affinity purification and on‐resin transthioesterification with (MESNa) over 66 h at 4 °C. After elution, the protein was further purified by semi‐preparative RP‐HPLC, and fractions were lyophilised (5 mg mL^−1^ culture medium). Ub_1–75_‐BocONH_2_K6‐SR was reconstituted by the addition of DMSO (38 μL) and then MQ water (155 μL), thus giving a final DMSO concentration of 20 % (*v*/*v*). Aminoacetaldehyde diethyl acetal (97 μL; 4 m adjusted to pH 8) was added to the solution followed by triethylamine (7 μL), thereby raising the solution to pH 9–10. The solution was vortexed briefly and incubated at 30 °C. The reaction was monitored by LCMS until completion. The resulting aminolysis protein product was purified by RP‐HPLC, and appropriate fractions were pooled and lyophilised (50 % yield).

**Generation of polyUb‐ox**: Lyophilised Ub_1–75_‐BocONH_2_K6‐acetal (2 mg) was reconstituted by the addition of DMSO followed by PBS (pH 7.4) to a concentration of 2 mg mL^−1^ (DMSO, 3 % *v*/*v*). The reconstituted solution (250 μL) was chilled, and cold TFA was added (final, 60 %) followed by incubation at 4 °C to RT for 2 h. Subsequent ice‐cold ether precipitation and air‐drying afforded polymerised Ub. The precipitated material was dissolved in NaH_2_PO_4_ (200 μL, 200 mm, pH 8) with GdnCl (6 m) then refolded by overnight dialysis (0.1–0.5 mL dialysis cassette, 3.5 KDa MWCO; Pierce) against PBS (2 L). SDS‐PAGE analysis (4–12 % Bis‐Tris gel, MES‐SDS, 200 V) was used to assess the extent of polymerisation. A control reaction was carried out with Ub_1–75_‐BocONH_2_K6‐acetal.

## Supporting information

As a service to our authors and readers, this journal provides supporting information supplied by the authors. Such materials are peer reviewed and may be re‐organized for online delivery, but are not copy‐edited or typeset. Technical support issues arising from supporting information (other than missing files) should be addressed to the authors.

Supplementary

## References

[1] D. Popovic , D. Vucic , I. Dikic , Nat. Med. 2014, 20, 1242–1253. 25375928 10.1038/nm.3739

[2] A. Hershko , A. Ciechanover , Annu. Rev. Biochem. 1998, 67, 425–479. 9759494 10.1146/annurev.biochem.67.1.425

[3] D. Komander , M. Rape , Annu. Rev. Biochem. 2012, 81, 203–229. 22524316 10.1146/annurev-biochem-060310-170328

[4] D. Komander , M. J. Clague , S. Urbé , Nat. Rev. Mol. Cell Biol. 2009, 10, 550–563. 19626045 10.1038/nrm2731

[5] T. Schneider , D. Schneider , D. Rösner , S. Malhotra , F. Mortensen , T. U. Mayer , M. Scheffner , A. Marx , Angew. Chem. Int. Ed. 2014, 53, 12925–12929; 10.1002/anie.201407192 25196034

[6a] B. Nanduri , A. E. Suvarnapunya , M. Venkatesan , M. J. Edelmann , Curr. Pharm. Des. 2013, 19, 3234–3247; 23151130 10.2174/1381612811319180008 PMC5485257

[6b] A. Pal , M. A. Young , N. J. Donato , Cancer Res. 2014, 74, 4955–4966; 25172841 10.1158/0008-5472.CAN-14-1211

[6c] M. E. Sowa , E. J. Bennett , S. P. Gygi , J. W. Harper , Cell 2009, 138, 389–403. 19615732 10.1016/j.cell.2009.04.042 PMC2716422

[7a] L. Yin , B. Krantz , N. S. Russell , S. Deshpande , K. D. Wilkinson , Biochemistry 2000, 39, 10001–10010; 10933821 10.1021/bi0007019

[7b] N. D. Weikart , H. D. Mootz , ChemBioChem 2010, 11, 774–777; 20209558 10.1002/cbic.200900738

[7c] S. Eger , M. Scheffner , A. Marx , M. Rubini , J. Am. Chem. Soc. 2010, 132, 16337–16339; 21033666 10.1021/ja1072838

[7d] S. Sommer , N. D. Weikart , A. Brockmeyer , P. Janning , H. D. Mootz , Angew. Chem. Int. Ed. 2011, 50, 9888–9892; 10.1002/anie.201102531 21898723

[7e] H. P. Hemantha , S. N. Bavikar , Y. Herman-Bachinsky , N. Haj-Yahya , S. Bondalapati , A. Ciechanover , A. Brik , J. Am. Chem. Soc. 2014, 136, 2665–2673. 24437386 10.1021/ja412594d

[8] Y. E. Lewis , T. Abeywardana , Y. H. Lin , A. Galesic , M. R. Pratt , ACS Chem. Biol. 2016, 11, 931–942. 26726734 10.1021/acschembio.5b01042 PMC6300141

[9a] M. Haj-Yahya , N. Eltarteer , S. Ohayon , E. Shema , E. Kotler , M. Oren , A. Brik , Angew. Chem. Int. Ed. 2012, 51, 11535–11539; 10.1002/anie.201205771 23065695

[9b] N. Haj-Yahya , M. Haj-Yahya , C. A. Castañeda , L. Spasser , H. P. Hemantha , M. Jbara , M. Penner , A. Ciechanover , D. Fushman , A. Brik , Angew. Chem. Int. Ed. 2013, 52, 11149–11153; 10.1002/anie.201306118 PMC3904769 24006204

[10] D. Flierman , G. J. van der Heden van Noort , R. Ekkebus , P. P. Geurink , T. E. T. Mevissen , M. K. Hospenthal , D. Komander , H. Ovaa , Cell Chem. Biol. 2016, 23, 472–482. 27066941 10.1016/j.chembiol.2016.03.009 PMC4850247

[11a] Y. Sato , A. Yoshikawa , A. Yamagata , H. Mimura , M. Yamashita , K. Ookata , O. Nureki , K. Iwai , M. Komada , S. Fukai , Nature 2008, 455, 358–362; 18758443 10.1038/nature07254

[11b] T. E. T. Mevissen , M. K. Hospenthal , P. P. Geurink , P. R. Elliott , M. Akutsu , N. Arnaudo , R. Ekkebus , Y. Kulathu , T. Wauer , F. El Oualid , S. M. V. Freund , H. Ovaa , D. Komander , Cell 2013, 154, 169–184. 23827681 10.1016/j.cell.2013.05.046 PMC3705208

[12] J. J. Sims , R. E. Cohen , Mol. Cell 2009, 33, 775–783. 19328070 10.1016/j.molcel.2009.02.011 PMC2709242

[13] S. Ulrich , D. Boturyn , A. Marra , O. Renaudet , P. Dumy , Chem. Eur. J. 2014, 20, 34–41. 24302514 10.1002/chem.201302426

[14] A. Shanmugham , A. Fish , M. P. A. Luna-Vargas , A. C. Faesen , F. El Oualid , T. K. Sixma , H. Ovaa , J. Am. Chem. Soc. 2010, 132, 8834–8835. 20540574 10.1021/ja101803s

[15a] B. Hao , W. Gong , T. K. Ferguson , C. M. James , J. A. Krzycki , M. K. Chan , Science 2002, 296, 1462–1466; 12029132 10.1126/science.1069556

[15b] G. Srinivasan , C. M. James , J. A. Krzycki , Science 2002, 296, 1459–1462; 12029131 10.1126/science.1069588

[15c] S. K. Blight , R. C. Larue , A. Mahapatra , D. G. Longstaff , E. Chang , G. Zhao , P. T. Kang , K. B. Green-Church , M. K. Chan , J. A. Krzycki , Nature 2004, 431, 333–335. 15329732 10.1038/nature02895

[16] D. P. Nguyen , M. M. Garcia Alai , P. B. Kapadnis , H. Neumann , J. W. Chin , J. Am. Chem. Soc. 2009, 131, 14194–14195. 19772323 10.1021/ja906603s

[17] S. Virdee , Y. Ye , D. P. Nguyen , D. Komander , J. W. Chin , Nat. Chem. Biol. 2010, 6, 750–757. 20802491 10.1038/nchembio.426

[18] T. Yanagisawa , R. Ishii , R. Fukunaga , T. Kobayashi , K. Sakamoto , S. Yokoyama , Chem. Biol. 2008, 15, 1187–1197. 19022179 10.1016/j.chembiol.2008.10.004

[19] S. Virdee , P. B. Kapadnis , T. Elliott , K. Lang , J. Madrzak , D. P. Nguyen , L. Riechmann , J. W. Chin , J. Am. Chem. Soc. 2011, 133, 10708–10711. 21710965 10.1021/ja202799r PMC3135006

[20] R. J. Mancini , R. C. Li , Z. P. Tolstyka , H. D. Maynard , Org. Biomol. Chem. 2009, 7, 4954–4959. 19907786 10.1039/b904195h PMC3086890

[21] A. Gautier , D. P. Nguyen , H. Lusic , W. An , A. Deiters , J. W. Chin , J. Am. Chem. Soc. 2010, 132, 4086–4088. 20218600 10.1021/ja910688s

[22] D. P. Nguyen , M. Mahesh , S. J. Elsässer , S. M. Hancock , C. Uttamapinant , J. W. Chin , J. Am. Chem. Soc. 2014, 136, 2240–2243. 24479649 10.1021/ja412191m PMC4333589

[23] K. D. Wilkinson , T. Gan-Erdene , N. Kolli , Methods Enzymol. 2005, 399, 37–51. 16338347 10.1016/S0076-6879(05)99003-4

[24] Y. Ye , M. Akutsu , F. Reyes-Turcu , R. I. Enchev , K. D. Wilkinson , D. Komander , EMBO Rep. 2011, 12, 350–357. 21399617 10.1038/embor.2011.17 PMC3077245

[25] M. H. Tatham , M.-C. Geoffroy , L. Shen , A. Plechanovova , N. Hattersley , E. G. Jaffray , J. J. Palvimo , R. T. Hay , Nat. Cell Biol. 2008, 10, 538–546. 18408734 10.1038/ncb1716

[26] J. S. Bett , M. S. Ritorto , R. Ewan , E. G. Jaffray , S. Virdee , J. W. Chin , A. Knebel , T. Kurz , M. Trost , M. H. Tatham , R. T. Hay , Biochem. J. 2015, 466, 489–498. 25489924 10.1042/BJ20141349 PMC4353193

[27] C. A. Castañeda , J. Liu , T. R. Kashyap , R. K. Singh , D. Fushman , T. A. Cropp , Chem. Commun. 2011, 47, 2026–2028. 10.1039/c0cc04868b PMC3190230 21212884

[28] A. Dirksen , T. M. Hackeng , P. E. Dawson , Angew. Chem. Int. Ed. 2006, 45, 7581–7584; 10.1002/anie.200602877 17051631

[29] W. P. Jencks , Prog. Phys. Org. Chem. 1964, 63–128.

[30] U. Hassiepen , U. Eidhoff , G. Meder , J.-F. Bulber , A. Hein , U. Bodendorf , E. Lorthiois , B. Martoglio , Anal. Biochem. 2007, 371, 201–207. 17869210 10.1016/j.ab.2007.07.034

[31] A. Hershko , I. A. Rose , Proc. Natl. Acad. Sci. USA 1987, 84, 1829–1833. 3031653 10.1073/pnas.84.7.1829 PMC304534

[32] J. D. Chodera , D. L. Mobley , Annu. Rev. Biophys. 2013, 42, 121–142. 23654303 10.1146/annurev-biophys-083012-130318 PMC4124006

[33] K. Newton , M. L. Matsumoto , I. E. Wertz , D. S. Kirkpatrick , J. R. Lill , J. Tan , D. Dugger , N. Gordon , S. S. Sidhu , F. A. Fellouse , L. Komuves , D. M. French , R. E. Ferrando , C. Lam , D. Compaan , C. Yu , I. Bosanac , S. G. Hymowitz , R. F. Kelley , V. M. Dixit , Cell 2008, 134, 668–678. 18724939 10.1016/j.cell.2008.07.039

[34] M. L. Matsumoto , K. C. Dong , C. Yu , L. Phu , X. Gao , R. N. Hannoush , S. G. Hymowitz , D. S. Kirkpatrick , V. M. Dixit , R. F. Kelley , J. Mol. Biol. 2012, 418, 134–144. 22227388 10.1016/j.jmb.2011.12.053

[35] J. A. Nathan , H. T. Kim , L. Ting , S. P. Gygi , A. L. Goldberg , EMBO J. 2013, 32, 552–565. 23314748 10.1038/emboj.2012.354 PMC3579138

[36a] V. H. Trang , E. M. Valkevich , S. Minami , Y.-C. Chen , Y. Ge , E. R. Strieter , Angew. Chem. Int. Ed. 2012, 51, 13085–13088; 10.1002/anie.201207171 PMC4083817 23161800

[36b] T. Moyal , S. N. Bavikar , S. V. Karthikeyan , H. P. Hemantha , A. Brik , J. Am. Chem. Soc. 2012, 134, 16085–16092. 22963682 10.1021/ja3078736

[37] L. Zhang , J. P. Tam , Anal. Biochem. 1996, 233, 87–93. 8789151 10.1006/abio.1996.0011

[38] A. G. van der Veen , H. L. Ploegh , Annu. Rev. Biochem. 2012, 81, 323–357. 22404627 10.1146/annurev-biochem-093010-153308

[39] H. Neumann , S. Y. Peak-Chew , J. W. Chin , Nat. Chem. Biol. 2008, 4, 232–234. 18278036 10.1038/nchembio.73

[40] P. Schmidt , L. Zhou , K. Tishinov , K. Zimmermann , D. Gillingham , Angew. Chem. Int. Ed. 2014, 53, 10928–10931; 10.1002/anie.201406132 25164607

[41] P. Schmidt , C. Stress , D. Gillingham , Chem. Sci. 2015, 6, 3329–3333. 10.1039/c5sc00921a PMC5656983 29142692

[42] K. Lang , J. W. Chin , Chem. Rev. 2014, 114, 4764–4806. 24655057 10.1021/cr400355w

[43] P. R. Chen , D. Groff , J. Guo , W. Ou , S. Cellitti , B. H. Geierstanger , P. G. Schultz , Angew. Chem. Int. Ed. 2009, 48, 4052–4055; 10.1002/anie.200900683 PMC2873846 19378306

[44a] I. S. Carrico , B. L. Carlson , C. R. Bertozzi , Nat. Chem. Biol. 2007, 3, 321–322; 17450134 10.1038/nchembio878

[44b] D. Rabuka , J. S. Rush , G. W. deHart , P. Wu , C. R. Bertozzi , Nat. Protoc. 2012, 7, 1052–1067. 22576105 10.1038/nprot.2012.045 PMC3498491

[45] W. Kabsch , Acta Crystallogr. Sect. D Biol. Crystallogr. 2010, 66, 125–132. 20124692 10.1107/S0907444909047337 PMC2815665

[46] P. D. Adams , P. V. Afonine , G. Bunkóczi , V. B. Chen , I. W. Davis , N. Echols , J. J. Headd , L.-W. Hung , G. J. Kapral , R. W. Grosse-Kunstleve , A. J. McCoy , N. W. Moriarty , R. Oeffner , R. J. Read , D. C. Richardson , J. S. Richardson , T. C. Terwilliger , P. H. Zwart , Acta Crystallogr. Sect. D Biol. Crystallogr. 2010, 66, 213–221. 20124702 10.1107/S0907444909052925 PMC2815670

[47] P. Emsley , B. Lohkamp , W. G. Scott , K. Cowtan , Acta Crystallogr. Sect. D Biol. Crystallogr. 2010, 66, 486–501. 20383002 10.1107/S0907444910007493 PMC2852313

