# Supplementary material for: Genetically Directed Production of Recombinant, Isosteric and Nonhydrolysable Ubiquitin Conjugates
Source: Chembiochem. 2016 Jun 27;17(15):1472–80. doi: 10.1002/cbic.201600138 (PMC5094518; doi:10.1002/cbic.201600138)
Supplement: Supplementary file 1 — Supplementary [file CBIC-17-1472-s001.pdf]

CHEMBIOCHEM

Supporting Information

Genetically Directed Production of Recombinant, Isosteric and Nonhydrolysable Ubiquitin Conjugates

Mathew Stanley and Satpal Virdee*[a]

cbic_201600138_sm_miscellaneous_information.pdf

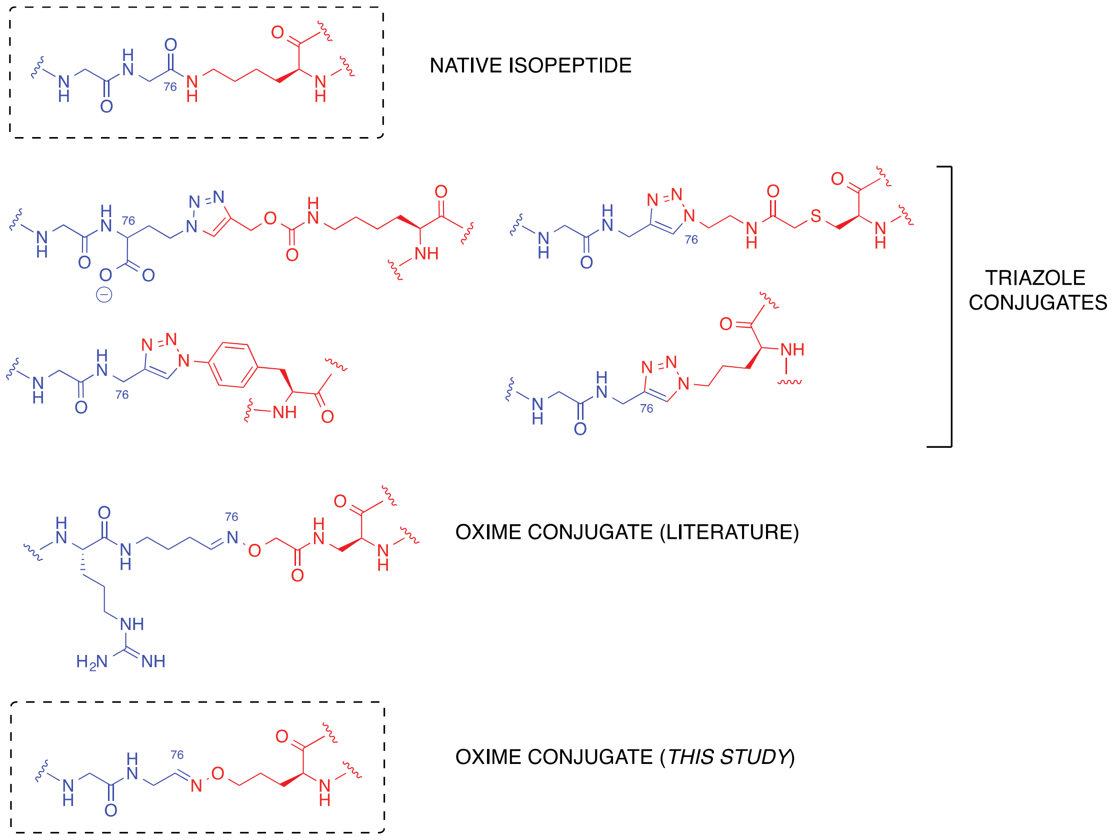

Supplementary Figure 1. Current methods for preparing non-hydrolyzable ubiquitin conjugates of recombinant origin have compromised isostery with native isopeptide-linked conjugates. Red represents the acceptor lysine residue from a protein substrate and blue corresponds to C-terminal Ub residues G75 and G76, or their surrogate due to design of the linkage. The oxime conjugate described in this study displays high isostery with the native isopeptide bond. Literature examples of oxime isosteres applied to Ub-conjugates typically introduce a potentially perturbing unnatural amide linkage within the lysine side chain and significantly deviate from the native peptide backbone.

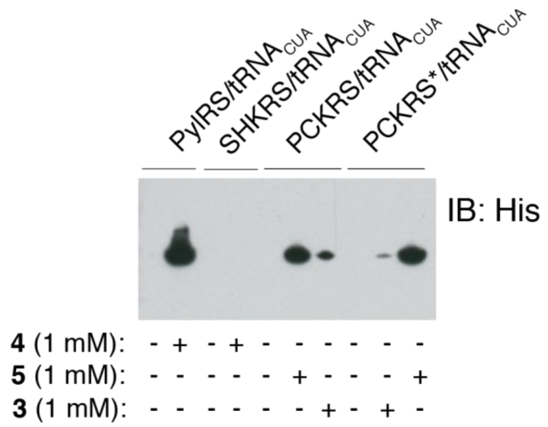

Supplementary Figure 2. Genetically directed incorporation of photocaged aminooxy amino acid 3 into sfGFP-TAG150-His (sfGFP). Photocaged amino acid **3** could be incorporated into superfolder GFP by the PCKRS/$tRNA_{CUA}$ pair. Compound **5** is photocaged lysine that was prepared as previously described[1].

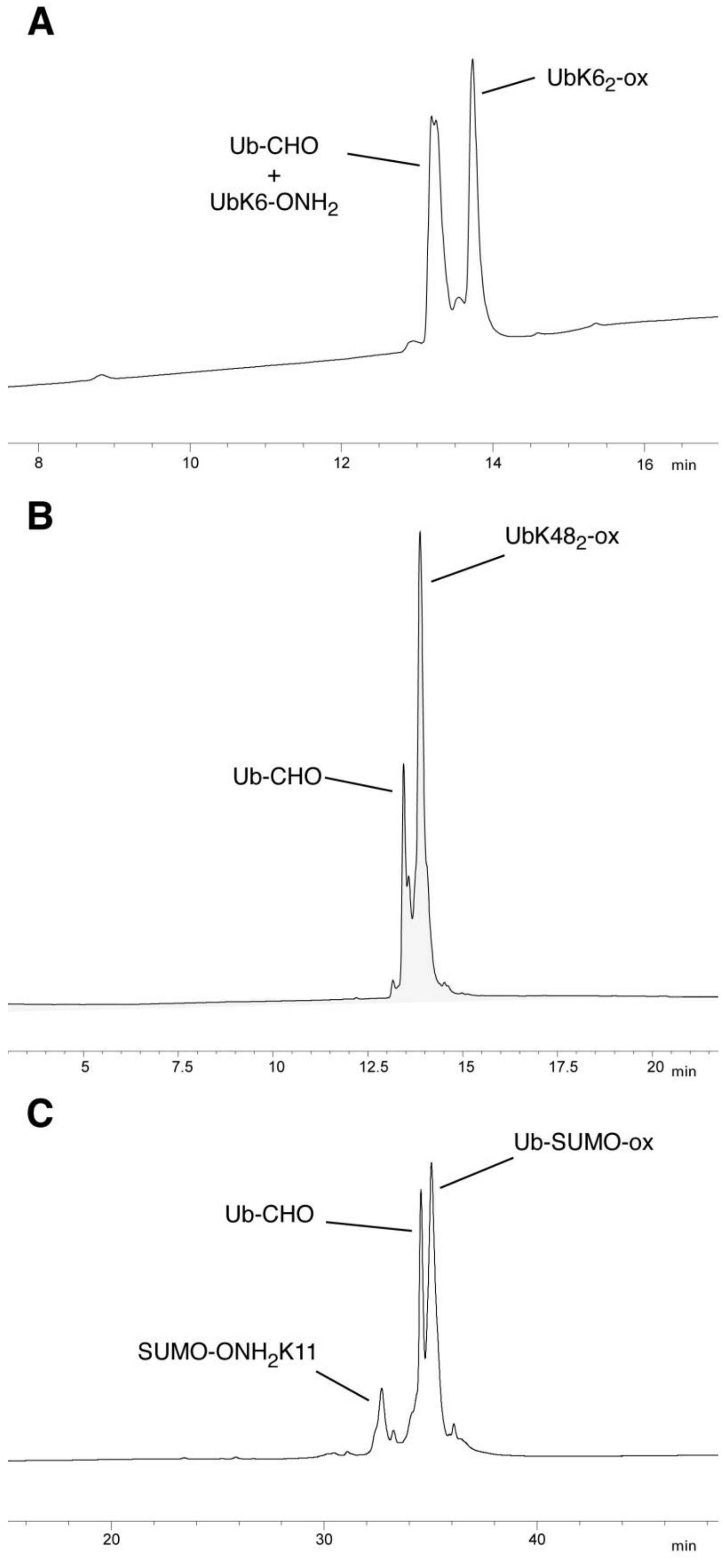
A
UbK6$_2$-ox
Ub-CHO
+
UbK6-ONH$_2$
8
10
12
14
16
min
B
UbK48$_2$-ox
Ub-CHO
5
7.5
10
12.5
15
17.5
20
min
C
Ub-SUMO-ox
Ub-CHO
SUMO-ONH$_2$K11
20
30
40
min

Supplementary Figure 3. Crude HPLC chromatograms of oxime ligation reactions to generate non-hydrolyzable Ub-conjugates (UV 214 nm). A) UbK6$_2$-ox B) UbK48$_2$-ox C) Ub-SUMO-ox.

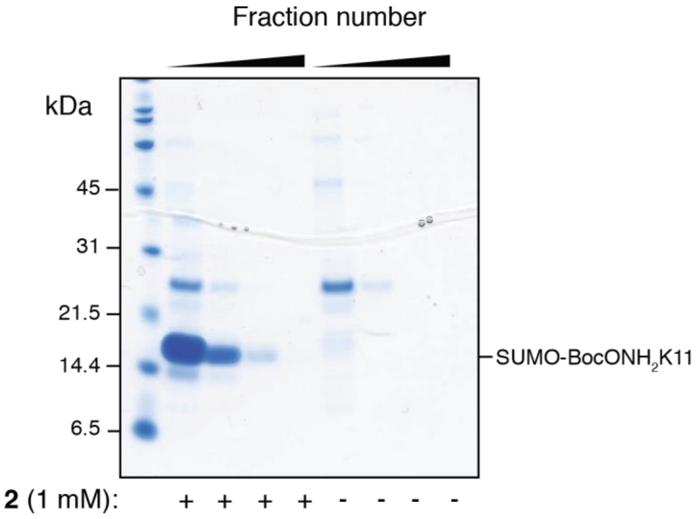

Supplementary Figure 4. Genetically directed incorporation of Boc-protected aminooxy amino acid 2 into SUMO2 containing a TAG codon at position 11 (SUMO-BocONH$_2$K11). *E. coli* was cultured in the presence and absence of **2**. Cells were lysed and then purification was carried out by Ni-NTA chromatography as SUMO2 contained a C-terminal Hisx6 tag. Full-length SUMO2 was only recovered in the presence of **2**.

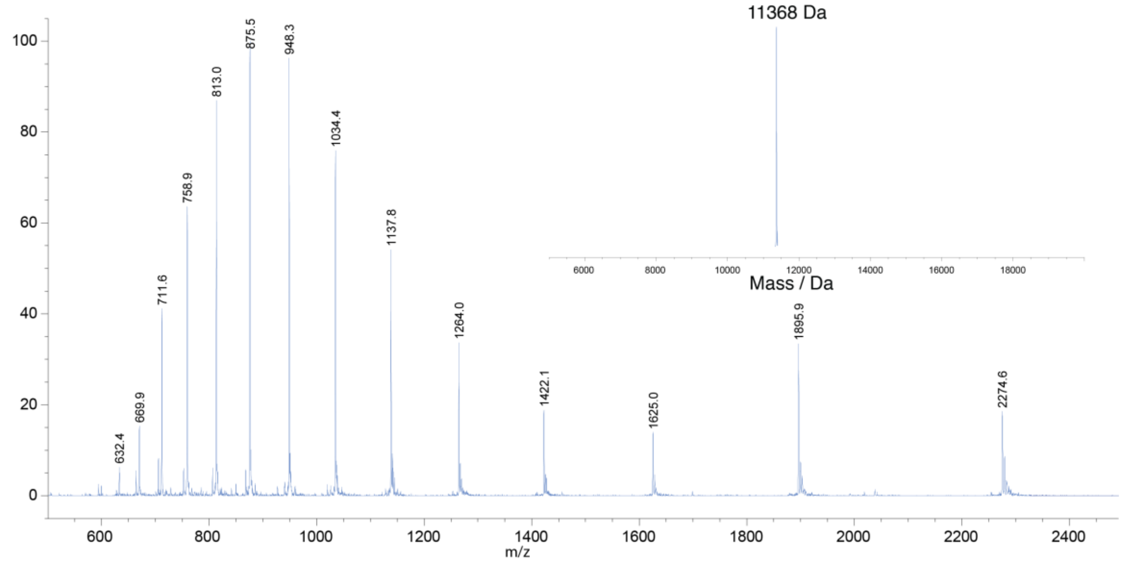

Supplementary Figure 5. LC-MS characterization of SUMO-BocONH_2K11. Observed mass = 11368 Da; theoretical mass = 11370.5 Da.

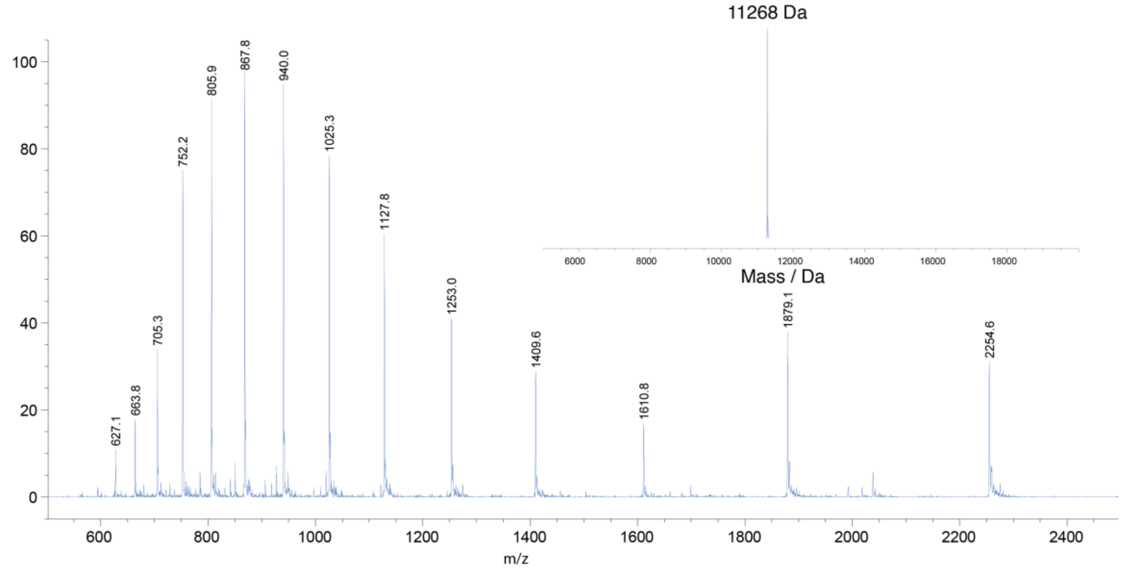

Supplementary Figure 6. LC-MS characterization of SUMO-ONH_2K11. SUMO-BocONH_2K11 was deprotected in the native state by the addition of 4 % TFA and incubation at 37 °C for 4 h. Observed mass = 11268 Da; theoretical mass = 11270.5 Da.

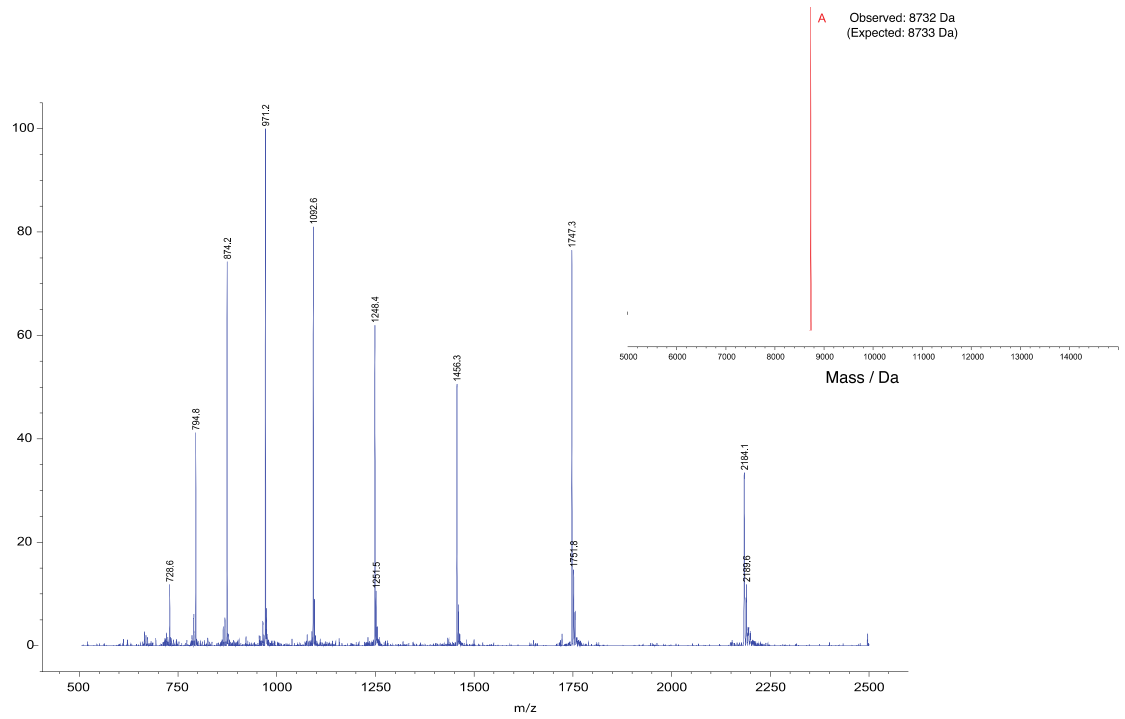

Supplementary Figure 7. LC-MS characterization of $Ub_{1\text{-}75}$-BocONH_2K6-SR

Observed mass = 8732 Da; theoretical mass = 8733 Da.

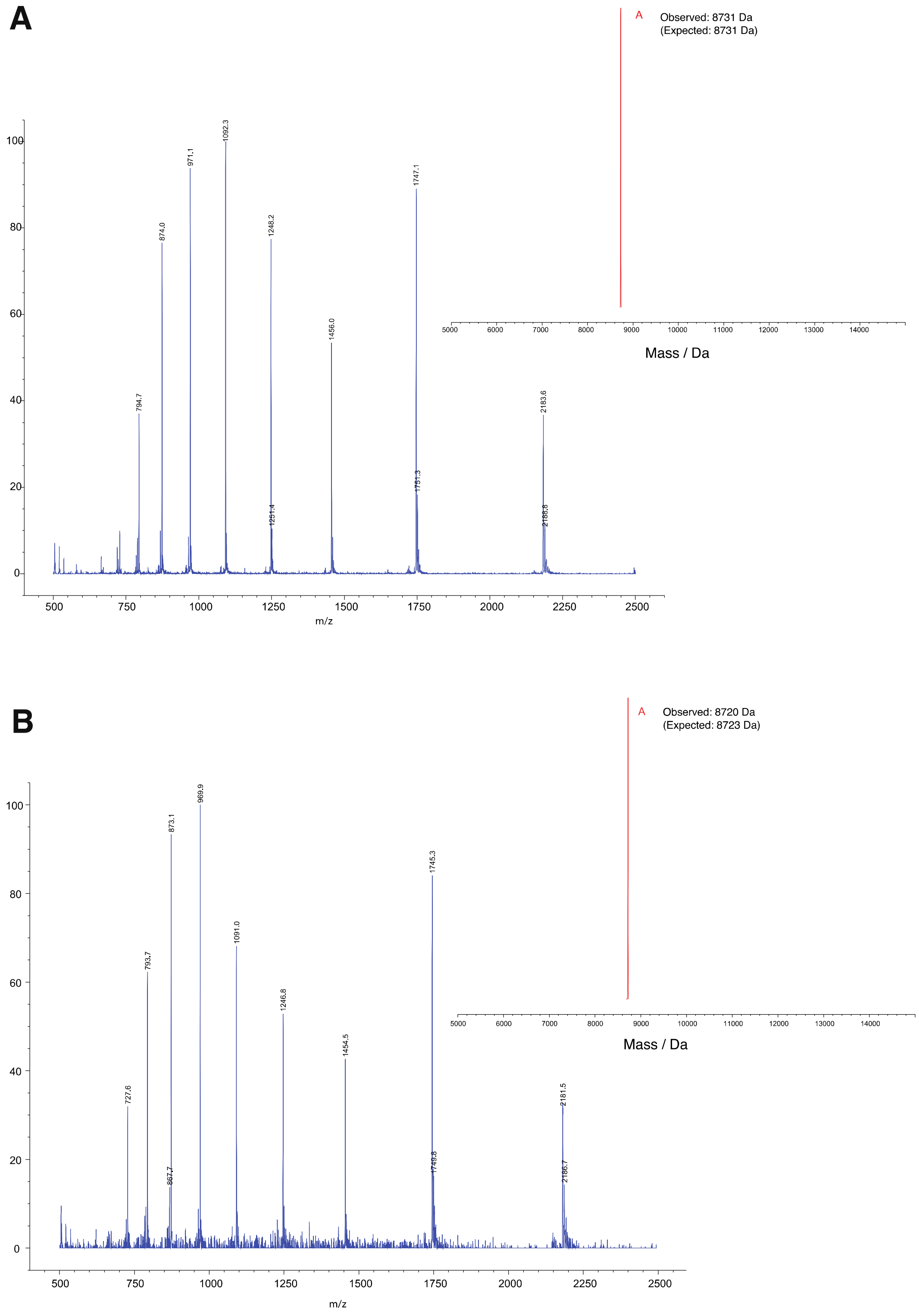

Supplementary Figure 8. LCMS characterization of $Ub_{1\text{-}75}$-BocK6-SR and $Ub_{1\text{-}75}$-BocK6-acetal. A) $Ub_{1\text{-}75}$-BocK6-SR, observed mass = 8731 Da; theoretical mass = 8731 Da. B) $Ub_{1\text{-}75}$-BocK6-acetal, observed mass = 8720 Da; theoretical mass = 8723 Da.

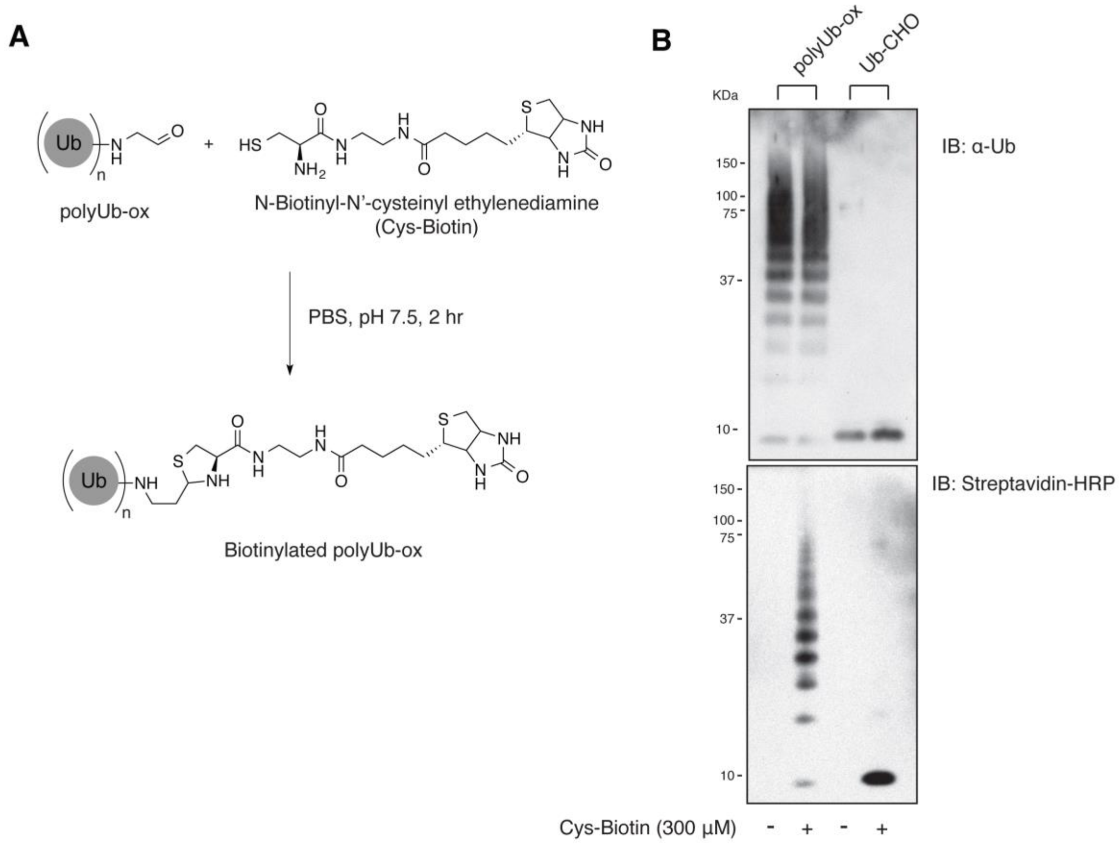

Supplementary Figure 9. Demonstration of polyUb-ox reporter group augmentation using commercially available N-Biotinyl-N'-cysteinyl ethylenediamine (Cys-Biotin) under physiological conditions. A) Thiazolidine formation between polyUb-ox and Cys-Biotin generates a covalent linkage between the Ub-species and the biotin reporter tag, rendering the non-hydrolyzable ubiquitin conjugates amenable to well-established avidin/streptavidin affinity enrichment experiments. B) (Upper blot) α-Ub blot (total Ub) indicates the presence of the polyUb-ox species and the non-polymerized Ub-CHO (derived from acidolysis of $Ub_{1\text{-}75}$-BocK6-acetal). (Lower blot) the presence of distinct bands in the streptavidin-HRP immunoblot is indicative of successful biotin labelling of both the polyUb-ox species and Ub-CHO, post-incubation with Cys-Biotin (300 μM). Samples untreated with Cys-Biotin are not immunoreactive.

	$UbK6_2$-ox
**Data collection**	
Space group	$P4_3321$
Cell dimensions	
a, *b*, *c* (Å)	103.975, 103.975, 103.975
α, β, γ (°)	90, 90, 90
Resolution (Å)	27.79 - 3.501 (3.626 - 3.501)
R_{merge}	0.05738 (0.5152)
$I / \sigma I$	6.77 (2.22)
Completeness (%)	99.0 (100.0)
Multiplicity	2.0 (2.0)
**Refinement**	
Resolution (Å)	27.79 - 3.501
No. reflections	2689 (254)
R_{work} / R_{free}	0.2716/0.3093
No. atoms	1096
Protein	1083
Ligand/ion	13
Water	0
B-factors	
Protein	126.24
Ligand/ion	178.39
R.m.s. deviations	
Bond lengths (Å)	0.022
Bond angles (°)	1.95

Supplementary Table 1. Crystallographic data collection and refinement statistics. Statistics for the highest-resolution shell are shown in parentheses.

General Methods

Chemicals were purchased from Sigma-Aldrich and used without further purification. All laboratory-reagent grade and analytical grade solvents were purchased from Fisher Scientific and Sigma-Aldrich. N-Biotinyl-N'-cysteinyl ethylenediamine TFA salt (Cys-Biotin) was purchased from Santa Cruz Biotechnology (sc-207989). Ultra pure water was obtained from a Millipore milliQ (MQ) Advantage system.

Semi-preparative HPLC was carried out on a Dionex Ultimate 3000 LC system with a 250 x 21.2 mm (ID) preparative column (Biobasic-4, 5 µm particle size, Thermo Scientific) and analytical HPLC was carried out with a 250 x 4.6 mm (ID) column (Biobasic-4, 5 µm particle size, Thermo Scientific). Flowrates were 10 mL min^{-1} and 1 ml min^{-1}, respectively. Buffer A

was H_2O containing 0.1 % trifluoroacetic acid (TFA) and buffer B was acetonitrile (ACN) containing 0.1 % TFA. Total protein mass was measured using electrospray ionization (ESI) on an Agilent Technologies 1200 single quadrupole LC-MS system fitted with a Max-Light Cartridge flow cell coupled to a 6130 Quadrupole spectrometer. The solvent system consisted of 0.05 % trifluoroacetic acid (TFA) in H_2O as buffer A, and 0.04 % TFA acid in acetonitrile (ACN) as buffer B. Protein UV absorbance was monitored at 214 and 280 nm. An Agilent ZORBAX 300SB-C3 5um, 2.1 x 150mm column was employed for proteins unless otherwise stated. Protein MS acquisition was carried out in positive ion mode and total protein masses were calculated by deconvolution within the MS Chemstation software (Agilent Technologies). Small molecule LC-MS was carried out using the Agilent system with an Agilent ZORBAX Eclipse Plus C18, 4.6 x 100 mm, 3.5 µm column. Variable wavelengths were used and MS acquisitions were carried out in positive and negative ion modes. NMR spectroscopic data were recorded on a 500 MHz Bruker instrument at room temperature. The following abbreviations are used to indicate the signal multiplicity: s (singlet), bs (broad singlet), d (doublet), t (triplet), q (quartet), m (multiplet).

Chemical Synthesis

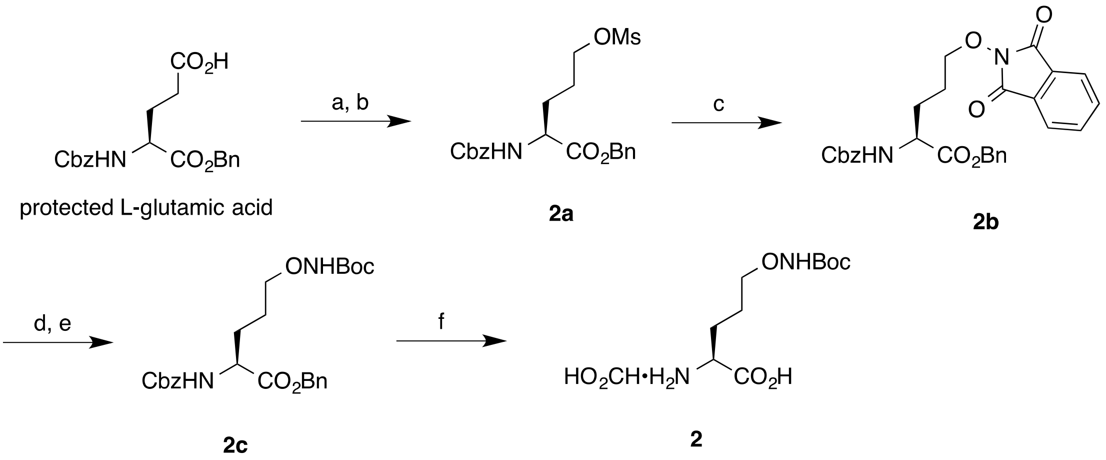

Supplementary Scheme 1. a) $ClCO_2Et$, 4-Methyl morpholine, THF followed by $NaBH_4$, MeOH b) MsCl, NEt_3, CH_2Cl_2. c) N-hydroxyphthalimide, DBU, DMF. d) *N*-methylhydrazine, DCM. e) Boc_2O, NEt_3, THF. f) Pd/C, MeOH/HCO_2H

Intermediates **2a – 2c** were synthesised according to literature procedures using protected L-glutamic acid as starting material[2] (**Supplementary Scheme 1.**).

***N*ε*-(t*-butyloxycarbonyl)-L-aminooxylysine, HCO_2H salt, 2**

Benzyl (S)-2-(((benzyloxy)carbonyl)amino)-5-(((tert-butoxycarbonyl)amino)oxy)pentanoate, **2c** (157 mg, 0.332 mmol) was suspended in anhydrous methanol (5 mL) supplemented with formic acid (4.4 % v/v) and placed under an inert atmosphere. Pd/C (10 % w/w) (315 mg) was also suspended in anhydrous methanol (5 mL) and placed under an inert atmosphere, followed by the addition of formic acid (final concentration; 4.4 % v/v). To the Pd/C suspension was added the methanolic solution. The resulting heterogeneous mixture was sonicated in an ultrasonic bath for 30 min at which time TLC analysis indicated product formation. Pd/C was removed by filtration over celite and the solution was dried *in vacuo*. The crude product was dissolved in water, passed through an Isolute Flash C18 SPE column (Biotage) and lyophillised to dryness giving a white powder **4** (90 mg, 95 %) (R_f: 0.1; $CHCl_3$/MeOH +1% AcOH).

1H NMR (500 Mhz, D_2O) δ 3.77 (1H, t, 6.2 Hz), 3.65 (1H, t, 6.1 Hz), 1.84 (2H, m), 1.61 (2H, m), 1.35 (9H, s). 13C NMR (126 MHz, D_2O) δ 174.41, 158.36, 83.37, 75.76, 54.41, 27.41, 27.08, 23.12. HR-MS: observed $[M+H]^+$ 249.1450 (calculated 249.1445).

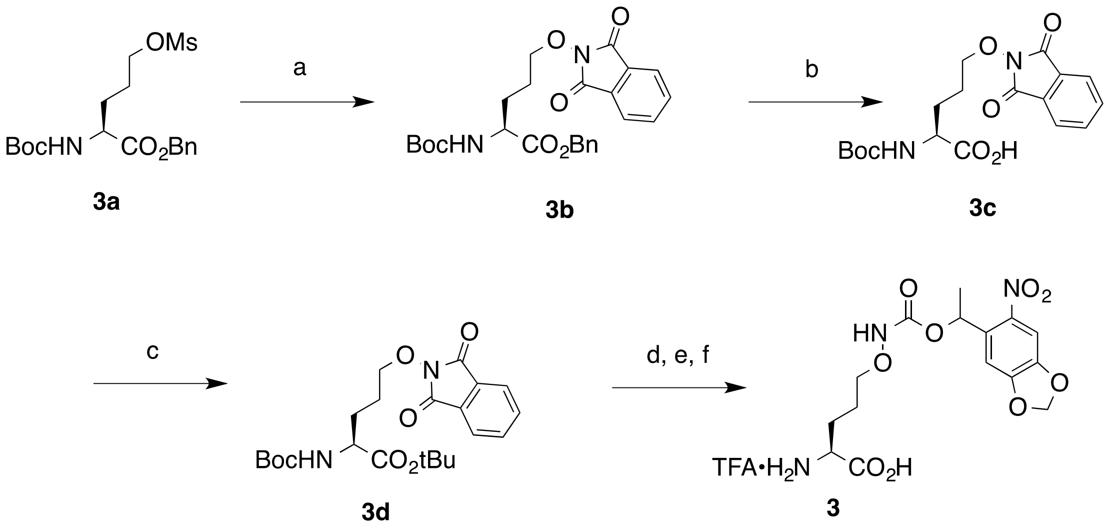

Supplementary Scheme 2. a) *N*-hydroxyphthalimide, DBU, DMF; b) MeOH, HCO_2H, Pd/C; c) DCM, *t*-butyl-2,2,2,-trichloroacetamidate; d) *N*-methylhydrazine, DCM; e) DCM/DMF/H_2O, $NaHCO_3$, NaOH, α-Methyl-6-nitropiperonylol succinimidyl carbonate; f) DCM/TFA, triethylsilane.

Intermediate 3b

N-Hydroxyphthalimide (2.86 g, 17.5 mmol) was dissolved in anhydrous DMF (17.5 mL) and was cooled to 0 °C. 1,8-Diazabicycloundec-7-ene (DBU) (2.6 mL, 17.5 mmol) was then added to the solution and was stirred for 30 min. benzyl (S)-2-((tert-butoxycarbonyl)amino)-5-((methylsulfonyl)oxy)pentanoate[3], **3a** (3.52 g, 8.77 mmol) was dissolved in anhydrous DMF (17.5 mL) and was added dropwise to the stirring solution. The solution was stirred for 5 h at which point the solvent was removed *in vacuo* at ambient temperature and was directly purified by flash chromatography (hexane/ethyl acetate; 3:1 → 2:1 → 1:1 → 1:2) to yield a clear oil which crystallized on cooling (2.89 g, 71% yield), R_f: 0.5 (hexane/ethyl acetate; 1:1). HR-MS: observed $[M+Na]^+$ 491.1789 (calculated 491.1794). 1H (500 MHz, $CDCl_3$) δ 7.80 – 7.85 (2H, m), 7.72 – 7.77 (2H, m), 7.29 – 7.39 (5H, m), 5.14 – 5.23 (3H, m), 4.36 – 4.43 (1H, m), 4.14 – 4.23 (2H, m), 2.06 – 2.17 (1H, m), 1.89 – 1.99

(1H, m), 1.72 – 1.85 (2H, m), 1.42 (9H, s). 13C NMR (126 MHz, CDCl3) δ 172.39, 163.62, 155.45, 135.39, 134.52, 128.91, 128.59, 128.38, 128.26, 123.55, 79.90, 77.58, 67.12, 53.25, 28.93, 28.32, 24.41.

Intermediate 3c

3b (407 mg, 0.869 mmol) was suspended in anhydrous methanol (4 mL) supplemented with formic acid (1 mL) and anhydrous dichloromethane (2 mL) and placed under an inert atmosphere. Pd/C (10 % w/w) (345 mg) was also suspended in anhydrous methanol (5 mL) and placed under an inert atmosphere, followed by the addition of formic acid (final concentration 4.4 % v/v). To the Pd/C suspension was added the methanolic solution of **3b**. The resulting heterogeneous mixture was sonicated in an ultrasonic bath for 2 h at which time TLC analysis indicated product formation. Pd/C was removed by filtration over celite and the solution was dried *in vacuo*. The crude product was purified by flash chromatography using a Reveleris® X2 flash chromatography system (Grace) (Reveleris® 12g silica column; EA:Hexane; 33% to 100% EA gradient elution) and evaporated dryness giving an oil (294 mg, 90 %). HR-MS: observed $[M+Na]^+$ 401.1319 (calculated 401.1325). 1H (400 MHz, $CDCl_3$) δ 10.0 (1H, bs), 7.78 – 7.83 (2H, m), 7.70 – 7.76 (2H m), 5.28 (1H, s, DCM), 4.33 – 4.41 (1H, m), 4.18 – 4.26 (2H, m), 2.10 – 2.21 (1H, m), 1.82 – 2.02 (3H, m), 1.42 (9H, s). 13C NMR (101 MHz, CDCl3) δ 176.55, 163.70, 156.64, 155.78, 134.54, 128.86, 123.58, 81.80, 80.20, 77.59, 54.22 (DCM), 53.00, 28.64, 28.29, 24.39.

Intermediate 3d

3c (900 mg, 2.38 mmol) was dissolved in anhydrous dichloromethane (10 mL) and cooled to 0 °C. Next, *t*-butyl-2,2,2,-trichloroacetamidate (950 μL, 5.30 mmol) was added to the solution and slowly stirred for 3 h and allowed to warm to room temperature. On

completion, sodium bicarbonate (50 mg) was added, the solution was filtered over celite and then the solvent removed *in vacuo*. The crude material was purified by flash chromatography using a Reveleris® X2 flash chromatography system (Grace) (Reveleris® 12g silica column; EA:Hexane; 10% to 40% 100% EA gradient elution) to yield a clear oil which crystallises on cooling (2.89 g, 71% yield), R_f: 0.5 (hexane/ethyl acetate; 1:1). HR-MS: observed $[M+Na]^+$ 491.1789 (calculated 491.1794). 1H (500 MHz, $CDCl_3$) δ 7.80 – 7.84 (2H, m), 7.72 – 7.76 (2H, m), 5.12 (2H, d, J = 7.5 Hz), 4.15 – 4.26 (3H, m), 1.98 – 2.09 (1H, m), 1.72 – 1.91 (3H, m), 1.46 (9H, s), 1.42 (9H, s). 13C NMR (126 MHz, CDCl3) δ 171.65, 163.57, 155.43, 134.47, 128.94, 123.50, 81.98, 79.62, 77.77, 77.23, 53.67, 29.20, 28.32, 28.00, 24.37.

(2*S*)-2-amino-5-((((1-(6-nitrobenzo[*d*][1,3]dioxol-5-yl)ethoxy)carbonyl)amino)oxy)pentanoic acid, TFA salt, 3

3d (725 mg, 1.67 mmol) was treated with methyl hydrazine in dichloromethane as reported[4], filtered over celite and used in subsequent steps without further purification. A portion of the crude reaction product (400 mg) was dissolved in DMF (3 mL) to which was added H_2O (2 mL). Sodium bicarbonate (275 mg, 3.27 mmol) was then added and the solution was stirred on ice. Whilst vigorously stirring, *α*-Methyl-6-nitropiperonylol succinimidyl carbonate[5] (507 mg, 1.44 mmol) in DMF (2 mL) was added to the solution, followed by 1 M NaOH (200 μL), adjusting the pH to ~9 (indicator paper). Care was taken to ensure exclusion of light as much as possible. After 10 min, dichloromethane (2 mL) was added and the stirring solution was taken off ice and warmed to room temperature. The solution was stirred vigorously for 10 h at which time the solution was neutralized to pH 7 (1M HCl) followed by removal of solvent *in vacuo*. The crude material was purified by flash chromatography (hexane/ethyl acetate; 2:1 → 1:1) to yield an amber coloured oil, R_f: 0.5 (hexane/ethyl acetate; 1:1). To this oil (150 mg, 0.309 mmol) was added 50% DCM/TFA (5

mL) and triethylsilane (74 μL, 0.463 mmol) under an inert atmosphere. The reaction was allowed to proceed for 14 h at which point the solvent was removed *in vacuo* without heating. The crude product was dissolved in water, passed through an Isolute Flash C18 SPE column (Biotage) and evaporated to dryness (70 mg, 62 % yield over 2 steps). HR-MS: observed $[M+Na]^+$ 386.1194 (calculated 386.1200).

1H NMR (500 Mhz, MeOH-D4) δ 7.26 (1H, s), 6.98 (1H, s), 6.11 (1H, q, 6.2 Hz), 6.03 (2H, s), 3.83 (3H, m), 1.94 (2H, m), 1.70 (2H, m), 1.45 (3H, m). 13C NMR (126 MHz, D_2O) δ 173.35, 157.41, 152.66, 147.03, 140.52, 134.82, 105.55, 104.68, 103.60, 75.83, 70.08, 53.66, 48.76, 26.83, 23.09, 20.90.

Supplementary Methods

Calibration curve

Due to the high UV absorbance of the aminooxy group, we could not use the empirically determined extinction coefficient of native ubiquitin[6] as a means to determine the concentration of Ub-oxime species spectrophotometrically.

Due to limited amounts of oxime-linked diubiquitin material available, generating stock solutions for generating a calibration curve using gravimetric determination was not suitable. To address this we assumed absorbance at 214 nm was approximately the same for native Ub_2 and Ub_2-oxime. Native Ub_2 solutions of defined concentration were analysed by LC-MS (5% to 95% acetonitrile (0.04% TFA) (flow rate; 0.3 mL min^{-1}) over 20 min and peaks were integrated and plotted against Ub_2 concentration. The peak area of Ub_2 at 214 nm was determined using the Agilent Chemsation software. Runs were repeated in duplicate.

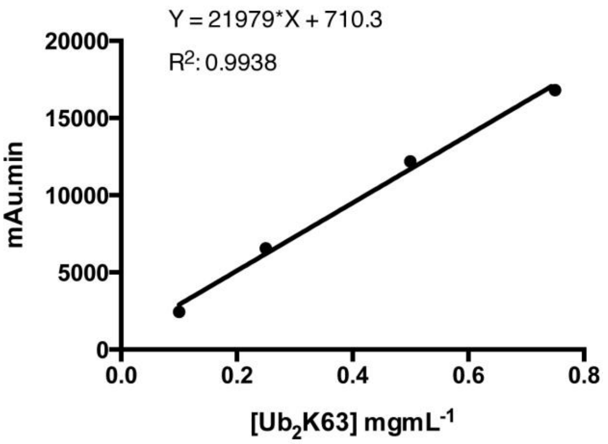

Plasmids, Protein Expression, and Purification

All DNA constructs were verified by DNA sequencing, which was performed by The Sequencing Service, School of Life Sciences, University of Dundee, using DYEnamic ET terminator chemistry (Amersham Biosciences) on Applied Biosystems automated DNA sequencers. DNA for bacterial protein expression was transformed into E. coli BL21-DE3 (Merck) unless otherwise stated.

Preparation of Ub-CHO

Lyophilized Ub_{1-75} thioester starting material was prepared as described for Ub_{1-76} [7]. However, the codon corresponding to ubiquitin residue Gly76 in the plasmid *pTXB1-Ub1-76*, was deleted by site-directed mutagenesis yielding plasmid *pTXB1-Ub1-75*. Ub_{1-75} thioester was then dissolved in denaturing buffer (200 mM Na_2HPO_4 pH 8.15, 6 M guanidinium chloride (GdnCl)) at a final concentration of 5 mg mL^{-1}. Aminoacetaldehyde diethyl acetal was then added at a final concentration of 250 mM and the reaction incubated at 37 °C for 4 h. Ub_{1-75} acetal was then purified by semi-preparative RP-HPLC applying a gradient of 20 – 50 % buffer B over 35 minutes. Fractions containing Ub_{1-75} acetal were determined by LC-MS and lyophilized.

Expression of Ub_{1-75}-BocONH$_2$K6-SR and Ub_{1-75}-BocK6-SR

ER2566 *E. coli.* cells (50 µL) (NEB) were doubly transformed with *plasmids pTXB1-Ub(TAG6)1-75* and *PylST-Y349W* and were recovered with S.O.C. medium (250 µL). The cells were incubated for 1 h at 37 °C and then LB medium (100 mL) containing ampicillin (100 µg mL^{-1}) and spectinomycin (50 µg mL^{-1}) were inoculated with the recovered cells (300 µL) and the culture was incubated overnight whilst shaking (200 rpm) at 37 °C. LB medium, pH 7.4 (2 x 500 mL) containing ampicillin (50 µg mL^{-1}) and spectinomycin (25 µg mL^{-1}) was inoculated with the overnight culture (25 mL / 500 mL culture) and incubated whilst shaking (200 rpm) at 37 °C. At O.D.600 ~ 0.6, **2** was added to the culture (to a final concentration of 1 mM) and the culture was incubated for a further 30 min in a 25 °C incubator. The cells were induced with IPTG (0.2 mM) and incubated for a further 5 h at 25 °C. The cells were harvested and suspended in 40 mL lysis buffer (20 mM Na_2HPO_4 pH 7.2, 200 mM NaCl, 1 mM EDTA). The cells were then lysed by sonication on ice and were clarified by centrifugation (39000 x g, 30 min). An empty XK 26/20 column was filled with chitin beads (10 mL) (NEB) and equilibrated with lysis buffer. At 4 °C the clarified lysate was loaded (flow rate; 0.7 mL min^{-1}) onto the column using an ÄKTA FPLC system. The column was then washed with lysis buffer (~ 100 mL) and equilibrated with 40 mL of cleavage buffer (20 mM Na_2HPO_4 pH 6, 200 mM NaCl, 100 mM MESNa, 1 mM EDTA). The flow was then stopped and the column incubated for 66 h at 4 °C, to allow cleavage of the ubiquitin thioester. Cleaved ubiquitin thioester, Ub_{1-75}-BocONH$_2$K6-SR, was eluted with elution buffer (20 mM Na_2HPO_4 pH 6, 200 mM NaCl, 1 mM EDTA). The appropriate fractions were determined by LCMS and were then pooled and concentrated to ~ 2.5 mL using an Amicon Ultra-15 centrifugal filter device (Millipore). The protein was then further purified by semi-preparative RP-HPLC (BioBasic-4; 72305-259270). A gradient of 20 % buffer A (MQ +

0.1% TFA) to 50 % buffer B (ACN + 0.1% TFA) was applied at a flow rate of 2.3 mL min^{-1} over 60 min. Appropriate fractions were confirmed by LCMS and were lyophilized, yielding ~5 mg L^{-1} of protein. Ub_{1-75}-BocK6-SR was prepared using the same method described above with the following amendments; 1. ER2566 *E. coli.* cells were doubly transformed with pTXB1-Ub(TAG6)1-75 and PylST; 2. Cells were supplemented with **4** (E1610, H-Lys(Boc)-OH, Bachem) in place of of **2**, as previously described.

Ubiquitin immunoblotting

Samples were resolved by SDS–PAGE (4–12 % NuPage gel, Invitrogen) with MES-SDS running buffer and transferred onto 0.2 μm PVDF membrane (GE Healthcare Life Science). Membranes were blocked with PBS-T buffer (PBS + 0.1 % Tween-20) containing 5 % (w/v) non-fat dried skimmed milk powder (PBS-TM) at room temperature for 1 h. Membranes were subsequently probed with the appropriate antibodies (see below) in PBS-T containing 5 % (w/v) Bovine Serum Albumin (BSA) or 5 % PBS-TM. Detection was performed using HRP-conjugated secondary antibodies where appropriate in PBS-TM for 1 h at room temperature at 1:10000 dilution. ECL Prime substrate (GE Life Sciences) was used for visualization in accordance with the manufacturers protocol.

For total Ub detection, samples were probed with 1:300 anti-Ub (Sigma U5379). Linkage specific antibody sources are outlined as follows; anti-K48Ub (#05-1307, Millipore), 1:1000; anti-K63Ub (#05-1313, Millipore), 1:1000; anti-Met1Ub is not commercially available and was obtained with special permission and used as described[8].

NMR Spectra

Compound 2 ^{1}H NMR

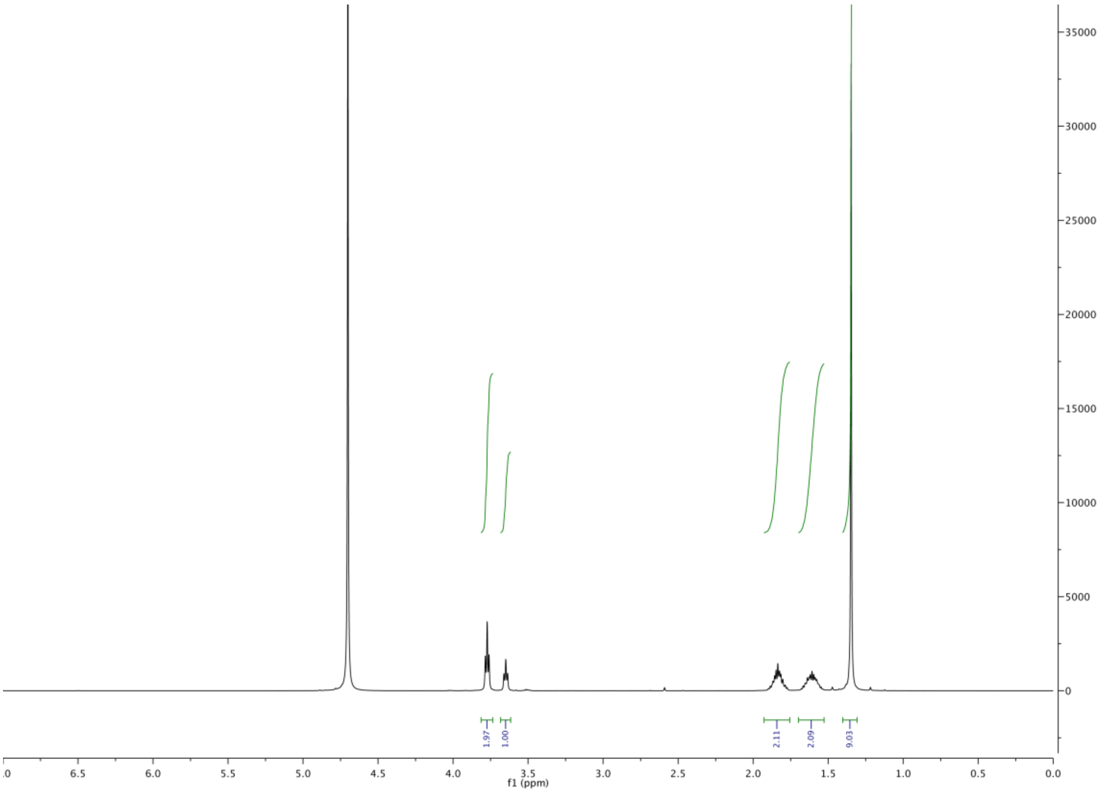

Compound 2 ^{13}C NMR

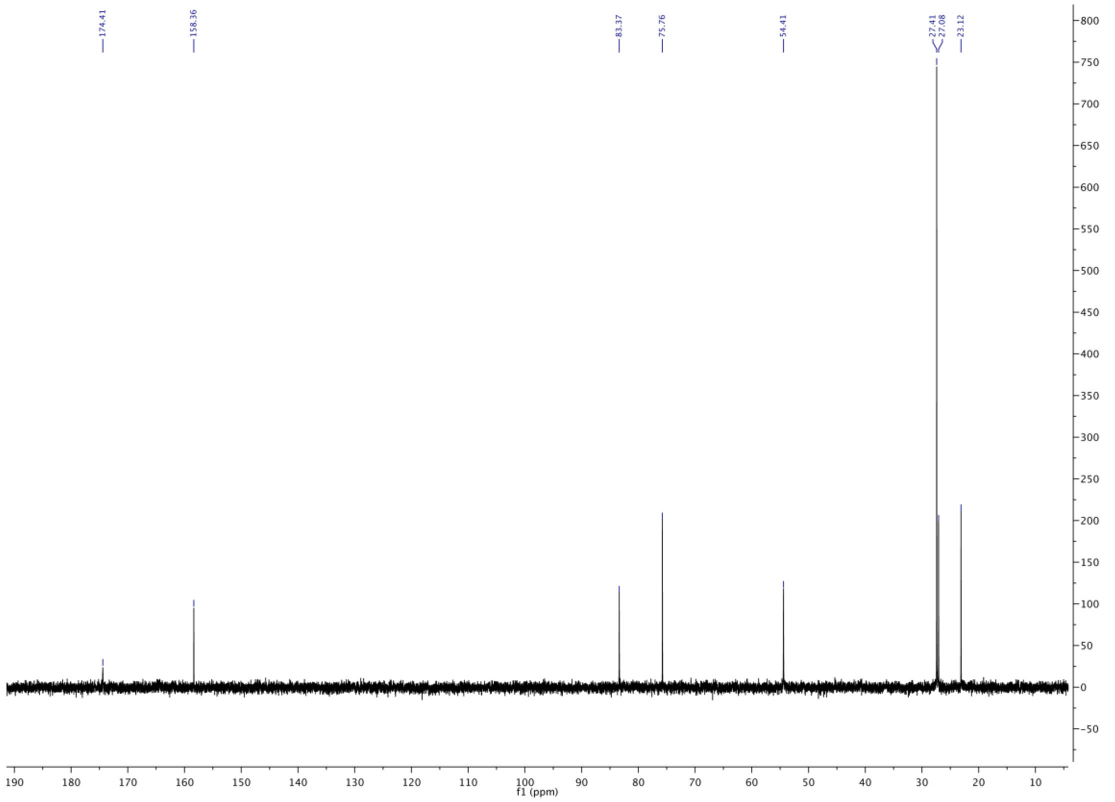

174.41
158.36
83.37
75.76
54.41
27.41
27.08
23.12
f1 (ppm)

Compound 3b ^{1}H NMR

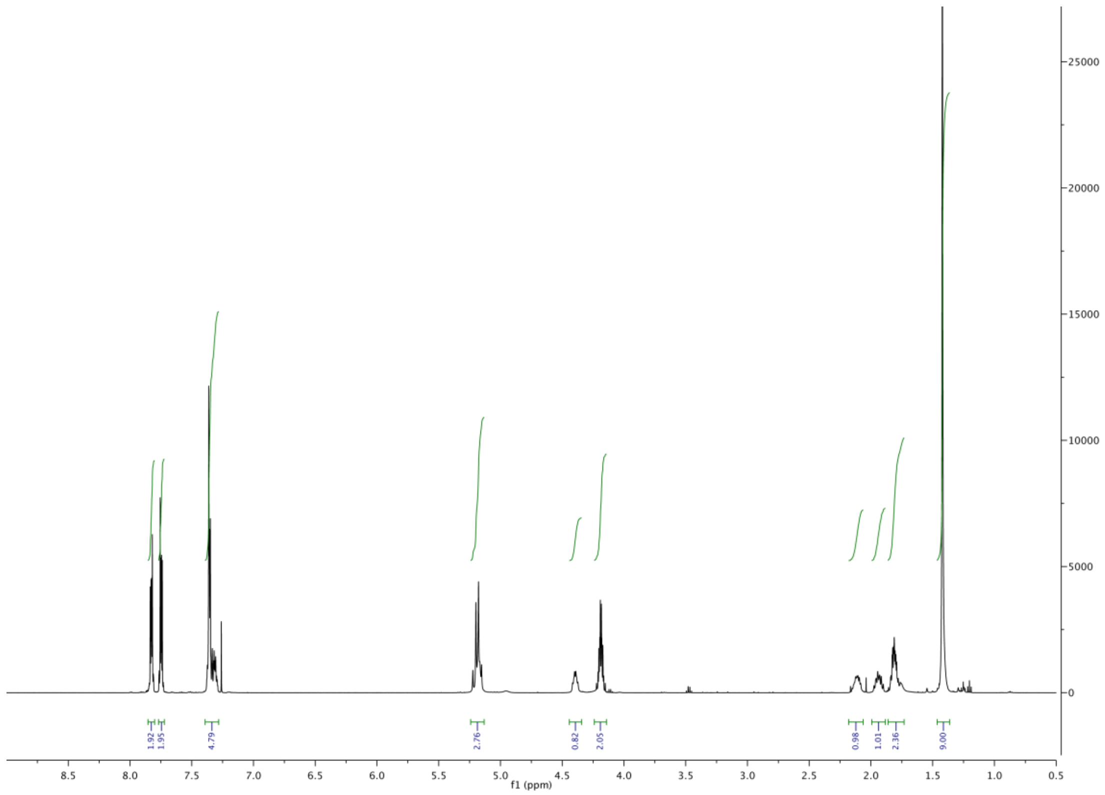

Compound 3b ^{13}C NMR

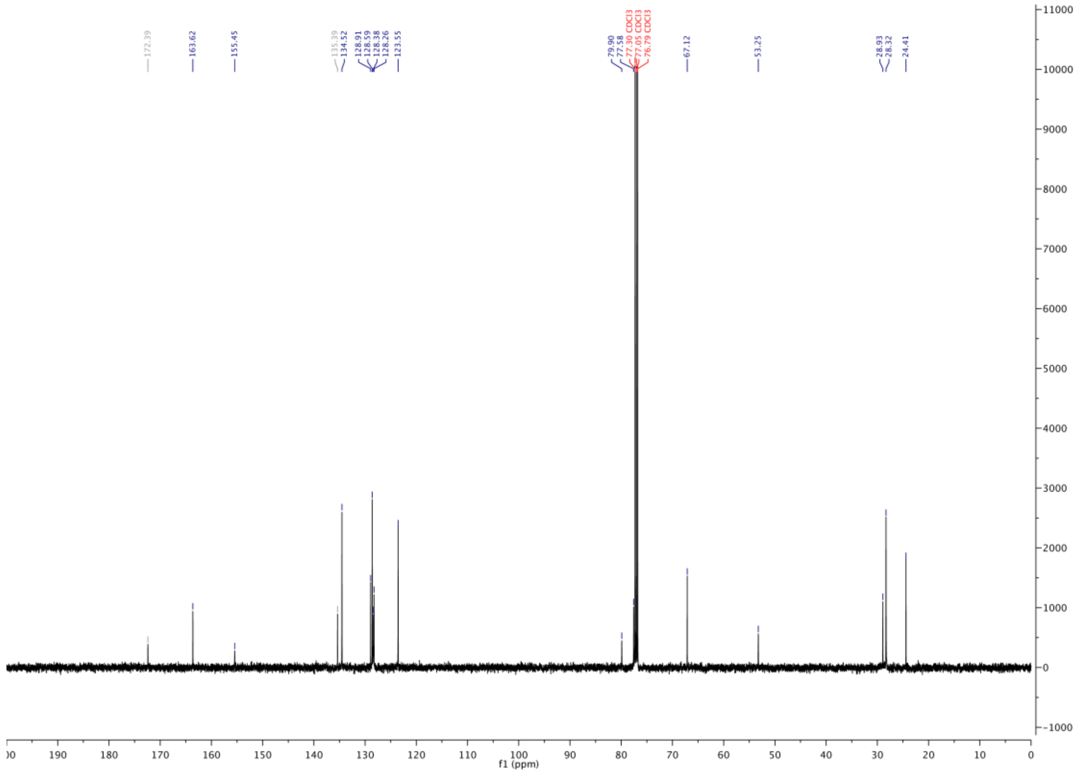

Compound 3c ^{1}H NMR

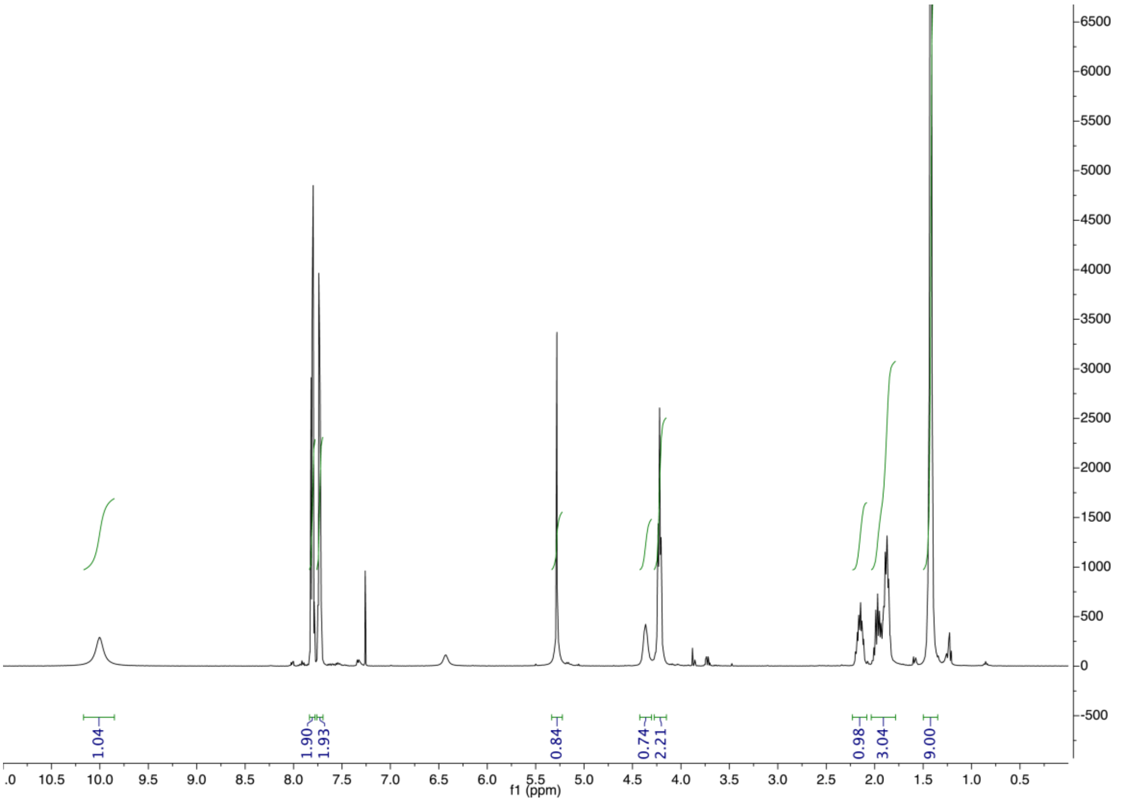

Compound 3c 13C NMR

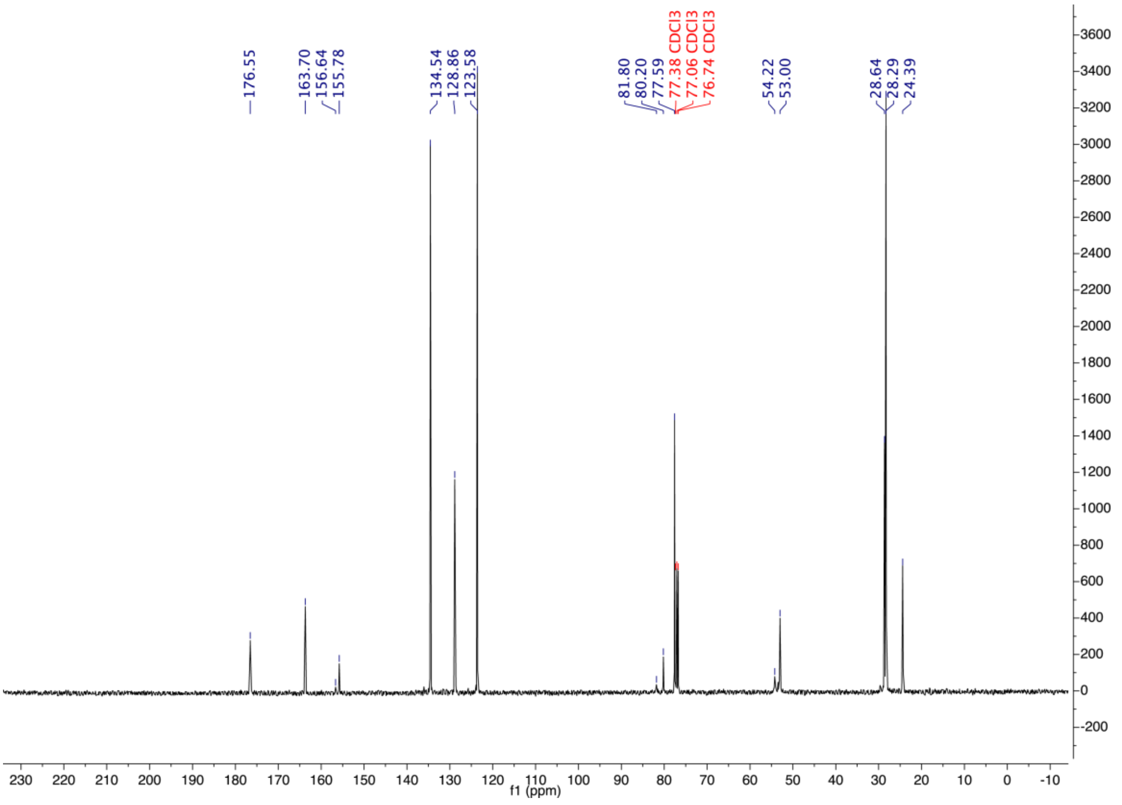

Compound 3d ^{1}H NMR

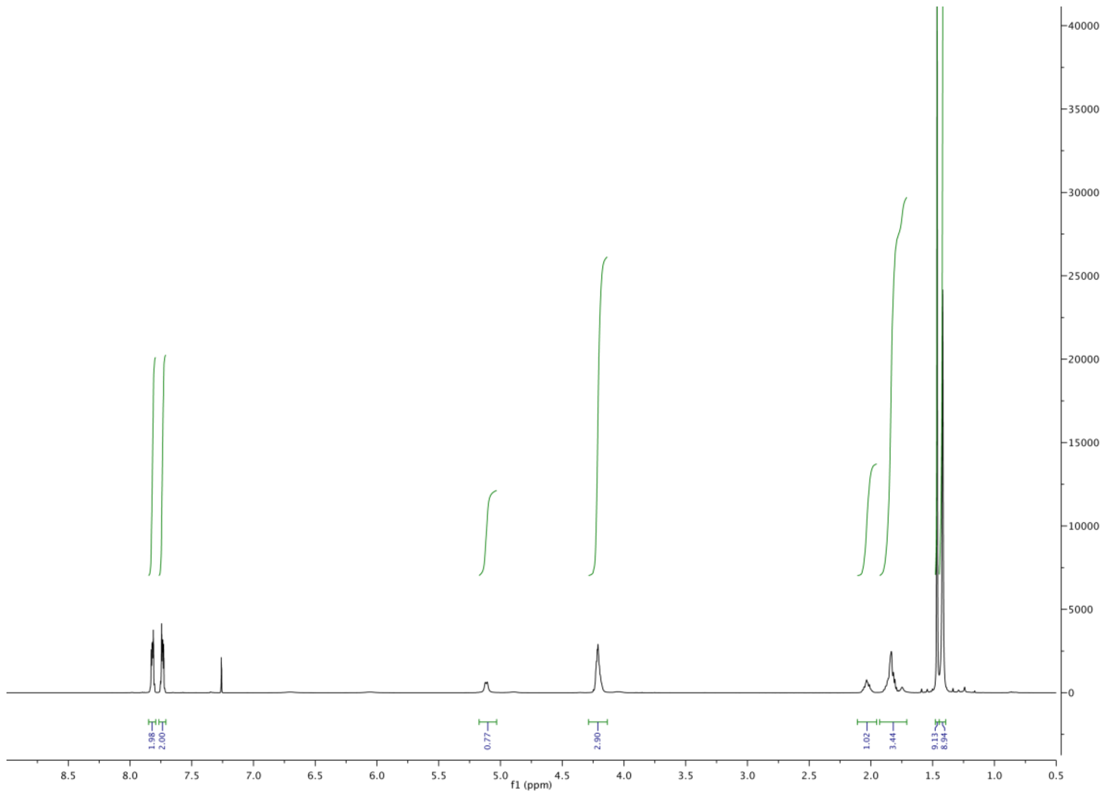

Compound 3d ^{13}C NMR

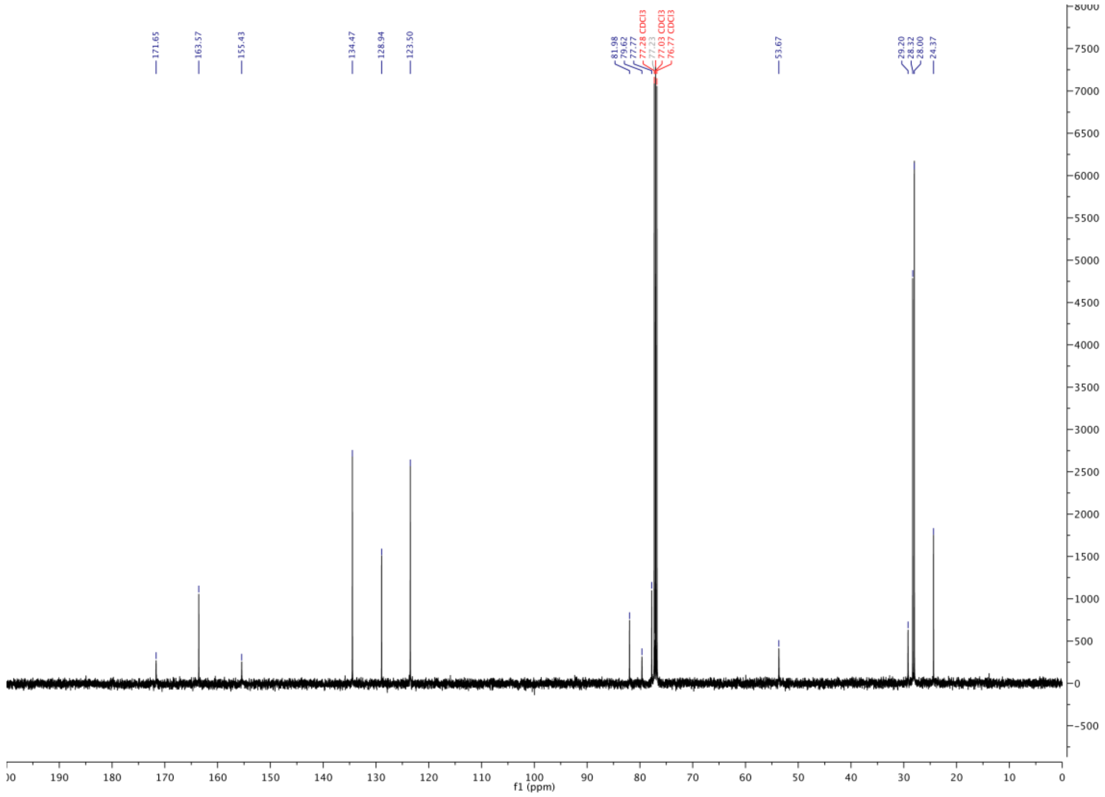

Compound 3 ^{1}H NMR

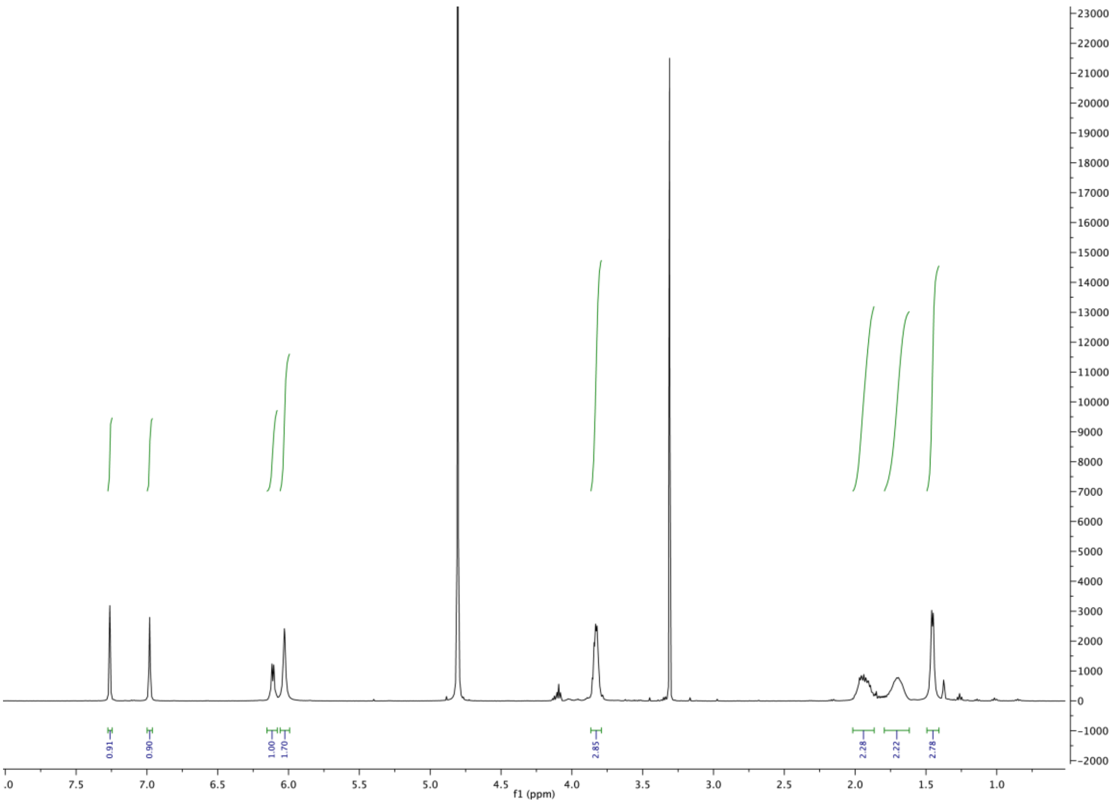

Compound 3 ^{13}C NMR

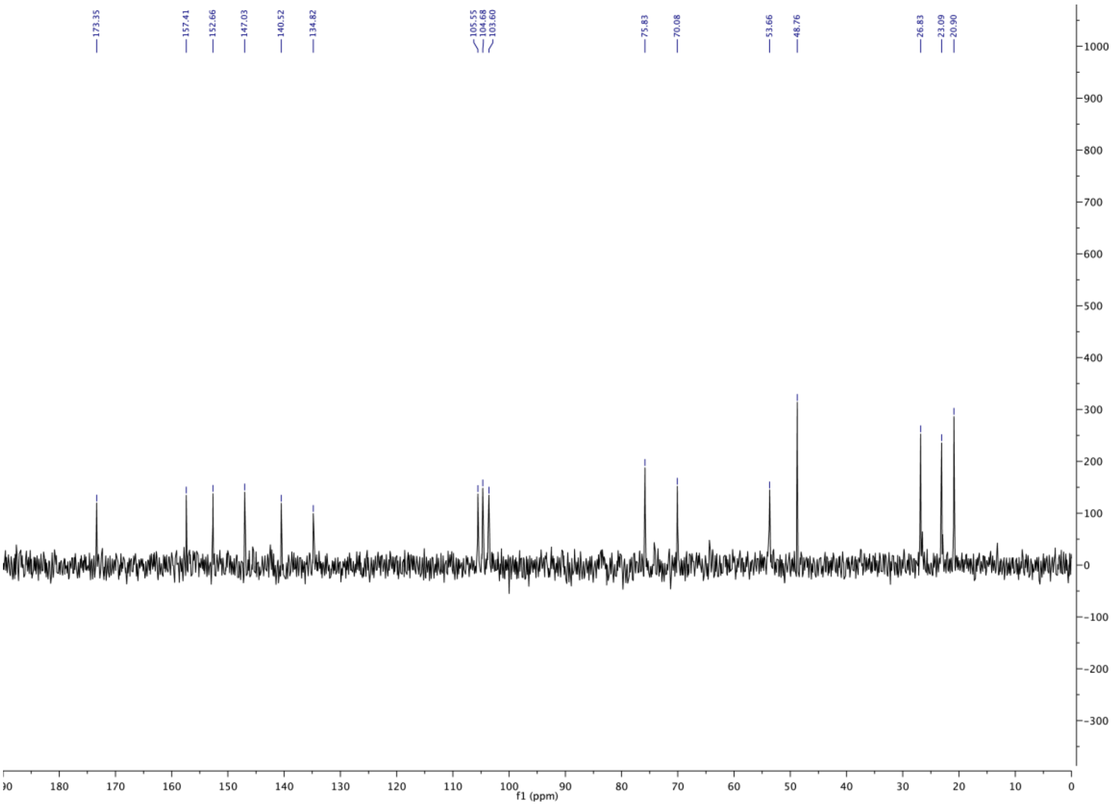

References

(1) Gautier, A.; Nguyen, D. P.; Lusic, H.; An, W.; Deiters, A.; Chin, J. W. *Journal of the American Chemical Society* **2010**, *132*, 4086.

(2) Liu, F.; Thomas, J.; Burke, T. R., Jr. *Synthesis (Stuttg)* **2008**, *15*, 2432.

(3) Roth, S.; Thomas, N. R. *Synlett* **2010**, 607.

(4) Liu, F.; Thomas, J.; Burke, T. R. *Synthesis* **2008**, *15*, 2432.

(5) Karginov, A. V.; Zou, Y.; Shirvanyants, D.; Kota, P.; Dokholyan, N. V.; Young, D. D.; Hahn, K. M.; Deiters, A. *Journal of the American Chemical Society* **2010**.

(6) Pickart, C. M.; Raasi, S. *Methods in enzymology* **2005**, *399*, 21.

(7) Virdee, S.; Ye, Y.; Nguyen, D. P.; Komander, D.; Chin, J. W. *Nature Chemical Biology* **2010**, *6*, 750.

(8) Matsumoto, M. L.; Dong, K. C.; Yu, C.; Phu, L.; Gao, X.; Hannoush, R. N.; Hymowitz, S. G.; Kirkpatrick, D. S.; Dixit, V. M.; Kelley, R. F. *J Mol Biol* **2012**, *418*, 134.
